# Inhibition of XPO-1 Mediated Nuclear Export through the Michael-Acceptor Character of Chalcones

**DOI:** 10.3390/ph14111131

**Published:** 2021-11-06

**Authors:** Marta Gargantilla, José López-Fernández, Maria-Jose Camarasa, Leentje Persoons, Dirk Daelemans, Eva-Maria Priego, María-Jesús Pérez-Pérez

**Affiliations:** 1 Instituto de Química Médica (IQM, CSIC) c/Juan de la Cierva 3, 28006 Madrid, Spain; mgargantilla@iqm.csic.es (M.G.); jose.lopez@iiq.csic.es (J.L.-F.); mj.camarasa@iqm.csic.es (M.-J.C.); 2 KU Leuven Department of Microbiology, Immunology and Transplantation, Laboratory of Virology and Chemotherapy, Rega Institute for Medical Research, KU Leuven, Herestraat 49, 3000 Leuven, Belgium; leentje.persoons@kuleuven.be (L.P.); dirk.daelemans@kuleuven.be (D.D.)

**Keywords:** chalcones, exportin-1, covalent binding, CovDock, anticancer activity

## Abstract

The nuclear export receptor exportin-1 (XPO1, CRM1) mediates the nuclear export of proteins that contain a leucine-rich nuclear export signal (NES) towards the cytoplasm. XPO1 is considered a relevant target in different human diseases, particularly in hematological malignancies, tumor resistance, inflammation, neurodegeneration and viral infections. Thus, its pharmacological inhibition is of significant therapeutic interest. The best inhibitors described so far (leptomycin B and SINE compounds) interact with XPO1 through a covalent interaction with Cys528 located in the NES-binding cleft of XPO1. Based on the well-established feature of chalcone derivatives to react with thiol groups via hetero-Michael addition reactions, we have synthesized two series of chalcones. Their capacity to react with thiol groups was tested by incubation with GSH to afford the hetero-Michael adducts that evolved backwards to the initial chalcone through a retro-Michael reaction, supporting that the covalent interaction with thiols could be reversible. The chalcone derivatives were evaluated in antiproliferative assays against a panel of cancer cell lines and as XPO1 inhibitors, and a good correlation was observed with the results obtained in both assays. Moreover, no inhibition of the cargo export was observed when the two prototype chalcones **9** and **10** were tested against a XPO1-mutated Jurkat cell line (XPO1C528S), highlighting the importance of the Cys at the NES-binding cleft for inhibition. Finally, their interaction at the molecular level at the NES-binding cleft was studied by applying the computational tool CovDock.

## 1. Introduction

Exportin-1 (XPO1, also known as chromosome region maintenance 1, CRM1) is the best-characterized nuclear transporter that mediates the traffic of high molecular weight molecules (i.e., proteins or RNA) from the nucleus to the cytoplasm [1,2]. Aberrant XPO1 function is implicated in different diseases, including different types of cancers, inflammation, neurodegeneration and viral infections [3,4,5,6,7,8]. In the nucleus, exportin-1 recognizes specific leucine-rich peptide stretches, known as nuclear export signals (NES) in cargo proteins, and forms a ternary complex with RanGTP. When this exportin-RanGTP-cargo complex reaches the cytoplasm, GTP is hydrolyzed to GDP, disrupting the ternary complex and releasing the cargo [2].

XPO1 has been found to be overexpressed in a variety of solid tumors and hematologic cancers, and in many cases, elevated XPO1 levels have been correlated to poor prognosis [7,9]. Pharmacological inhibition of XPO1 has been considered an appealing anticancer strategy [2,4,5,10]. Indeed, XPO1 cargo proteins include many tumor suppressors and cell growth regulators, such as p53, Topo2, FOXOs, etc., [7,8], and XPO1 inhibition has been shown to restore nuclear localization and function of these tumor suppressors, leading to apoptosis of the cancer cells [11].

The most potent XPO1 inhibitors described, either from natural or synthetic origin, are α,β-unsaturated carbonyl compounds that bind into the NES-binding cleft of XPO1 through covalent interactions with Cys528 [2,4,10,12,13]. These inhibitors prevent the interaction of cargo proteins with XPO1 and, hence, block cargo export to the cytoplasm [14]. Only recently, a non-covalent XPO1 inhibitor has been described [15], while a first report on allosteric inhibitors with moderate affinity has been published [16].

**Figure 1:**
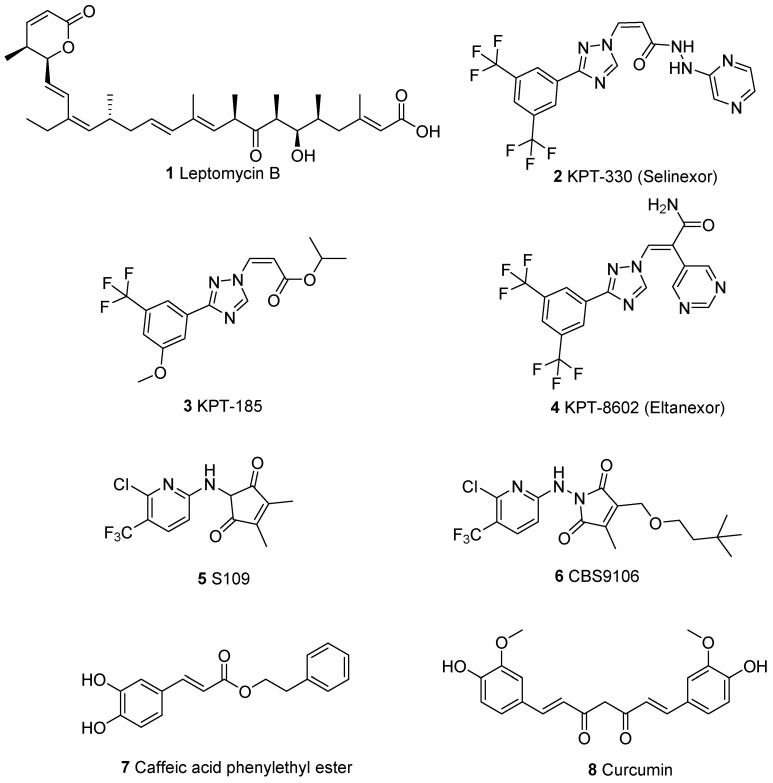
Chemical structures of several XPO1 inhibitors.

The first XPO1 inhibitor clinically tested was the natural product leptomycin B (**1**, Figure 1). However, clinical trials were discontinued due to severe cytotoxicity [17], probably associated with irreversible inhibition. Among synthetic compounds, the *N*-azolylacrylate compounds [12,13], also known as selective inhibitors of nuclear export (SINE) exemplified by KPT-330 (selinexor) (**2**), KPT-185 (**3**) or the second generation compound KPT-8602 (eltanexor) (**4**) (Figure 1), have been described as very slowly reversible inhibitors [12,18,19,20]. Indeed, the recent FDA approval of selinexor (KPT-330, XPOVIO, **2**) for the treatment of patients with heavily pretreated relapsed or refractory multiple myeloma and diffuse B cell lymphoma supports the significance of XPO1 as a target in hematological malignancies [21]. Other interesting XPO1 inhibitors are the 1-(pyridin-2-ylamino)-1*H*-pyrrole-2,5-dione derivatives S109 (**5**) and CBS9106 (**6**) [22,23], with the latter being evaluated in Phase 1 clinical trials [24].

**Figure 2:**
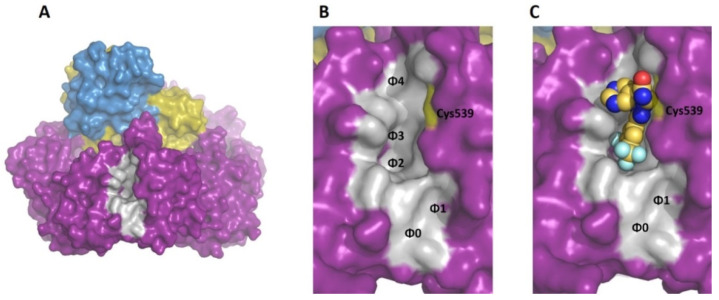
(**A**) Overall structure of the XPO1 protein (in deep purple), Ran (olive) and RanBP1 (sky blue). The NES-binding cleft is shown in grey. (**B**) Surface representation of the NES-binding cleft (grey) and the Φ0–Φ4 pockets. ScCys539 is shown in yellow and labelled. (**C**) Detailed view of KPT-8602 (yellow spheres, pdb id: 5jlj) inside of the NES-binding cleft.

The overall structure of XPO1 complexed with Ran and RanBP1 is shown in Figure 2A, where the NES-binding cleft is colored in grey. Structurally, this cleft contains five hydrophobic pockets (named Φ0–Φ4, Figure 2B) that lodge the hydrophobic key residues of the cargo protein. Cys528 (which corresponds to Cys539 in *Saccharomyces cerevisiae* in the X-ray structure) is located between the Φ3 and Φ4 pockets. While leptomycin B occupies almost all Φ pockets [17], the KPT compounds exemplified by KPT-8602 (Figure 2C) only occupy a small part of the NES-binding cleft [25], demonstrating that XPO1 inhibition can be accomplished by only occupying part of the hydrophobic cleft. By analysing the binding mode of KPT-8602, it can be concluded that an aromatic hydrophobic core is located below the Cys-reactive residue, while more polar substituents (a pyrimidinyl ring in KPT-8602) can be lodged over the Cys in the proximities of the Φ4 pocket.

**Figure 3:**
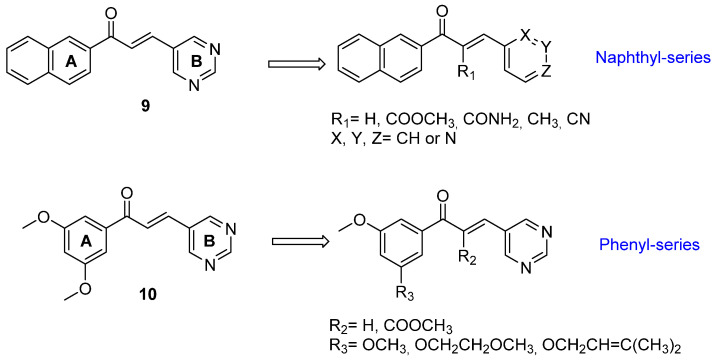
Chalcones synthesized and tested in this study as XPO1 inhibitors.

Based on these precedents, we envisioned the chalcone scaffold as a suitable α,β-unsaturated carbonyl construct for XPO1 inhibition by interaction with Cys528 based on their ability to behave as Michael acceptors. Indeed, quite similar α,β-unsaturated carbonyl compounds such as caffeic acid phenylethyl ester (**7**, Figure 1) or curcumin (**8**) have been described as XPO1 inhibitors. Moreover, since chalcones can undergo a retro-Michael reaction, the expected inhibition should be reversible. Reversible covalent inhibitors may have some advantages versus their irreversible counterparts, such as the possibility to tune the residence time and/or to avoid the irreversible inhibition of off-targets [26], while as a disadvantage they may show lower potency. Compounds **9** and **10** (Figure 3) were proposed as prototypes, so that ring A should be lodged below the Cys where the covalent interaction takes place, while the pyrimidine (ring B) should be located closer to the Φ4 pocket. The XPO1 inhibition obtained with these compounds triggered us to explore closely related structural analogues (Figure 3). Thus, their synthesis, antiproliferative activity against a panel of cancer cell lines, XPO1 inhibition and docking studies at the NES-binding cleft of XPO1 are here described.

## 2. Results and Discussion

### 2.1. Synthesis

**Scheme 1:**
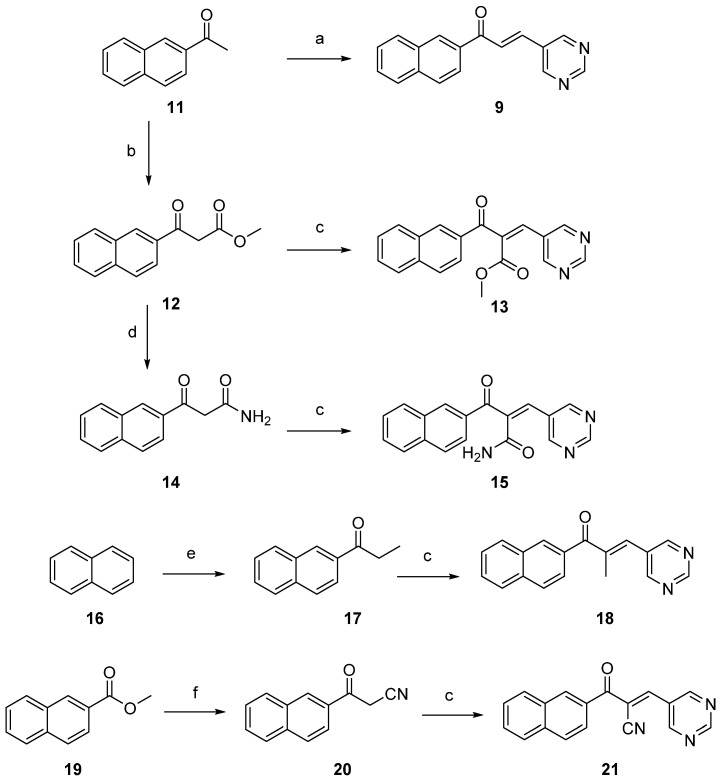
Reagents and conditions: (a) pyrimidine-5-carbaldehyde, Ba(OH)_2_, methanol/water, rt, 2 h, 55% yield; (b) as described in [28]: dimethyl carbonate, NaH, 1,4-dioxane, 70 °C, 3.5 h; (c) pyrimidine-5-carbaldehyde, piperidine, AcOH, 70–100 °C, 2–48 h, 30–40% yield; (d) NH_3_, 1,4-dioxane, 110 °C, 16 h, 34% yield; (e) as described in [30]: propionyl chloride, AlCl_3_, 1,2-DCE, rt, 16 h; (f) as described in [32]: acetonitrile, NaH, toluene, 110 °C, 16 h.

It has been described by performing kinetic measurements in α-X-2′3,4,4’-tetramethylchalcones that the nature of the substituent at position α of the chalcone affects their reactivity as electrophiles versus thiol groups [27]. In such series, the α-COOEt substituent led to a similar reactivity as that of the α-H chalcone; the α-CH_3_ chalcone was poorly electrophilic, while the α-CN chalcone was the most electrophilic. Thus, in the naphthyl series (Figure 3), the synthesis of the chalcones where R_1_ = H, COOCH_3_, CH_3_ or CN was accomplished. The reaction of 1-(naphthalen-2-yl)ethan-1-one (**11**) with pyrimidine-5-carbaldehyde in the presence of Ba(OH)_2_ afforded α-H chalcone **9** in a 55% yield (Scheme 1). The ketone **11** reacted with dimethyl carbonate, as described, to provide the methyl 3-oxopropanoate **12 [28]**, whose reaction with pyrimidine-5-carbaldehyde resulted in the α-COOCH_3_ chalcone **13**. Reaction of the ester **12** with NH_3_/dioxane at 110 °C [29] led to the amide **14** that was further transformed into the chalcone **15**. On the other hand, reaction of naphthalene **16** with propionyl chloride under Friedel–Crafts acylation conditions, as described [30], provided the 2-acyl derivative **17,** together with a small proportion of its 1-isomer [31]. This mixture of isomers reacted with pyrimidine-5-carbaldehyde to yield the α-CH_3_ chalcone **18** (30%). Finally, the synthesis of the α-CN derivative was addressed by the reaction of 2-methylnaphthoate (**19**) with acetonitrile to provide 3-oxopropanenitrile **20** [32], which was further transformed into the α-CN chalcone **21**. Compound **21** proved to be very unstable and readily decomposed in phosphate-buffered solution (PBS), hampering its biological evaluation.

**Scheme 2:**
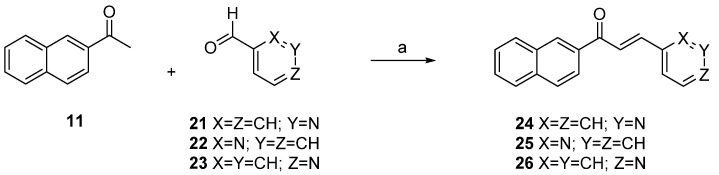
Reagents and conditions: (a) Ba(OH)_2_, methanol/water, rt, 2–7 h, 32–73% yield.

A second set of modifications involved the incorporation as ring B of different pyridines instead of the 5-pyrimidinyl of the prototype **9**. To this end, the ketone **11** was reacted with different aldehydes (**21**–**23**) in the presence of Ba(OH)_2_, using a mixture of methanol and water at rt, to yield the chalcones **24**–**26** (Scheme 2).

**Scheme 3:**
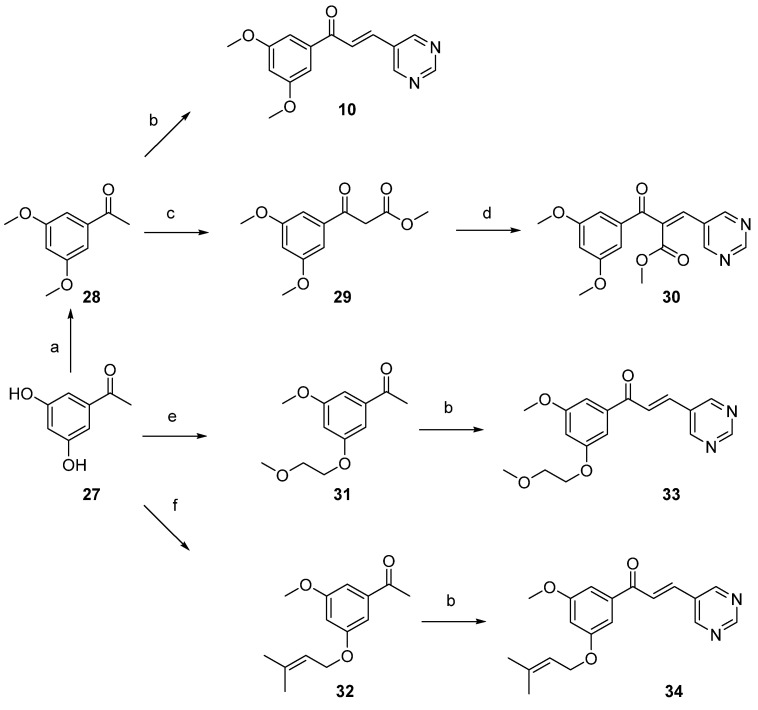
Reagents and conditions: (a) as described in [33]: MeI, Cs_2_CO_3_, DMF, 80 °C, 1 h; (b) pyrimidine-5-carbaldehyde, Ba(OH)_2_, methanol/water, rt, 16 h, 32–64% yield; (c) as described in [34]: dimethyl carbonate, NaH, 1,4-dioxane, 70 °C, 3 h; (d) pyrimidine-5-carbaldehyde, piperidine, AcOH, 70 °C, 16 h, 39% yield; (e) (i) as described in [35]: MeI, Cs_2_CO_3_, DMF, rt, 16 h, (ii) 2-bromoethylmethyl ether, Cs_2_CO_3_, DMF, 80 °C, 1 h, 77% yield; (f) (i) as described in [35]: MeI, Cs_2_CO_3_, DMF, rt, 16 h; (ii) 3,3-dimethylallyl bromide, Cs_2_CO_3_, DMF, 80 °C, 1 h, 75% yield.

In parallel, we synthesized the 3,5-dimethoxyphenyl chalcone **10** (Scheme 3) by reaction of the 1-(3,5-dihydroxyphenyl)ethan-1-one **27** with methyl iodide to provide the ketone **28** [33], whose reaction with pyrimidine-5-carbaldehyde afforded the chalcone **10** (64% yield). Additionally, in this case, the α-COOCH_3_ chalcone was synthesized through the transformation of the ketone **28** into the methylpropionate **29** [34], and further reaction of this methyl ester with pyrimidine-5-carbaldehyde afforded the α-COOCH_3_ chalcone **30**. On the other hand, as will be later shown in the molecular modeling studies, one of the methoxy groups of the chalcone **10** seems to be pointing towards the lower part of the NES-binding cleft that lodges the aliphatic chain of leptomycin B. Thus, one of the methoxy groups in the chalcone **10** was replaced by either a 2-methoxyethoxy or a 3,3-dimethylallyloxy group. To this end, the 3,5-dihydroxyacetophenone **27** was transformed in 2 steps into the corresponding ketones **31** and **32**, whose reaction with pyrimidine-5-carbaldehyde afforded the chalcones **33** and **34** in a 32% and 41% yield, respectively.

For the α-H chalcones (**9**, **10**, **24**–**26**, **33**, **34**), the configuration of the double bond was easily assigned as E, as expected, based on the J_H2-H3_ value (15–16 Hz) in their ^1^H-NMR spectra [36]. The configuration of the α-substituted chalcones (**13**, **15**, **18**, **21**, **30**) was also assigned as E, based on previous reports [37,38].

### 2.2. Incubation with GSH

In order to determine if these chalcones could react with thiol groups through a hetero-Michael addition reaction, a few selected compounds (**9**, **10**, **13** and **18**) were incubated with glutathione (GSH). After different incubation times, the reactions were quenched by adding 5,5′-dithiobis(2-nitrobenzoic acid) (DTNB, Ellman´s reagent) [39,40] (1:1 ratio with respect to the initial concentration of GSH). The course of the reaction was followed by HPLC, adapting reported procedures [41,42]. The prototype α-H chalcones **9** and **10** quickly reacted with GSH to provide the Michael addition products, as shown by HPLC-MS. These adducts underwent a retro-Michael addition reaction, generating the parent chalcone, demonstrating the reversibility of the reaction (see Figures S1 and S2). The α-COOCH_3_ chalcone **13** also provided the Michael adducts with GSH after a short reaction time, while longer incubations revealed that the adducts reverted to the parent chalcone (Figure S3). However, under the same incubation conditions with GSH, the α-CH_3_ chalcone **18** remained almost unaltered even after 4 h of incubation, and only a very small proportion of the addition products was detected (Figure S4). As already mentioned, the α-CN chalcone **21** could not even be tested due to instability in PBS, suggesting a very high electrophilic character. Thus, although this assay is only qualitative, the tendency observed among these chalcones nicely fits the reactivity described for α-X-2´3,4,4´-tetramethylchalcones [27].

### 2.3. Biological Results

#### 2.3.1. Antiproliferative Activity

The synthesized compounds were tested for their antiproliferative activity against a panel of cancer cell lines using KPT-330 as a reference compound (Table 1).

The two prototype compounds **9** and **10** inhibited proliferation against all cell lines tested at single digit µM IC_50_ values, being between 5- to 30-fold less potent than the reference compound KPT-330. The α-substituted chalcones in the naphthyl series with an ester (compound **13**) or methyl (compound **18**) group were almost inactive in the proliferation assays, while the α-CONH_2_ chalcone **15** was slightly less active than the parent chalcone **9**. When the 5-pyrimidinyl ring in **9** was replaced by different pyridines (pyridin-3-yl in **24**, pyridin-2-yl in **25** or pyridin-4-yl in **26**), the IC_50_ vales were maintained in the low µM range. Concerning the phenyl series, the α-COOCH_3_ chalcone **30** was considerably less active than the α-H chalcone **10**. Moreover, replacement of one of the methoxy groups in compound **10** by longer ethers was compatible with antiproliferative activity, although up to a 20-fold increase in IC_50_ values was obtained for certain cell lines.

#### 2.3.2. XPO1 Inhibition Studies

In order to determine if the observed antiproliferative activity was caused by XPO1 inhibition, the compounds were assayed in a reporter cell line based on the subcellular localization of a XPO1-dependent GFP reporter cargo protein [13]. Inhibition of XPO1-mediated nuclear export of the reporter is evident by its nuclear accumulation, which can be visualized and quantified compared to the untreated control, where the GFP protein localizes in the cytoplasm. Using this reporter cell line, the capacity of the synthesized chalcones to inhibit XPO1-mediated nuclear export was determined (Table 2).

The prototype chalcones **9** and **10** both in the naphthyl and phenyl series showed significant XPO1 inhibition, with IC_50_ values of 2.5 and 0.55 µM, respectively. Interestingly, among the naphthyl series, the chalcones substituted with an ester or a methyl group at the α-position of the double bond (compounds **13** and **18,** respectively) that were inactive in the antiproliferative activity assays, were also inactive against XPO1. On the other hand, the α-CONH_2_ chalcone **15** or compounds with a pyridinyl ring (**24–26**) instead of the pyrimidinyl of the prototype **9**, which had shown significant antiproliferative activity, inhibited XPO1 with IC_50_ values around or below 10 µM. Among the phenyl series, the α-COOCH_3_ derivative **30** was almost inactive against XPO1, while the extended ethers **33** and **34** were active, particularly the 3,3-dimethylallyl derivative **34**, with an IC_50_ value of 1.59 µM.

**Figure 4:**
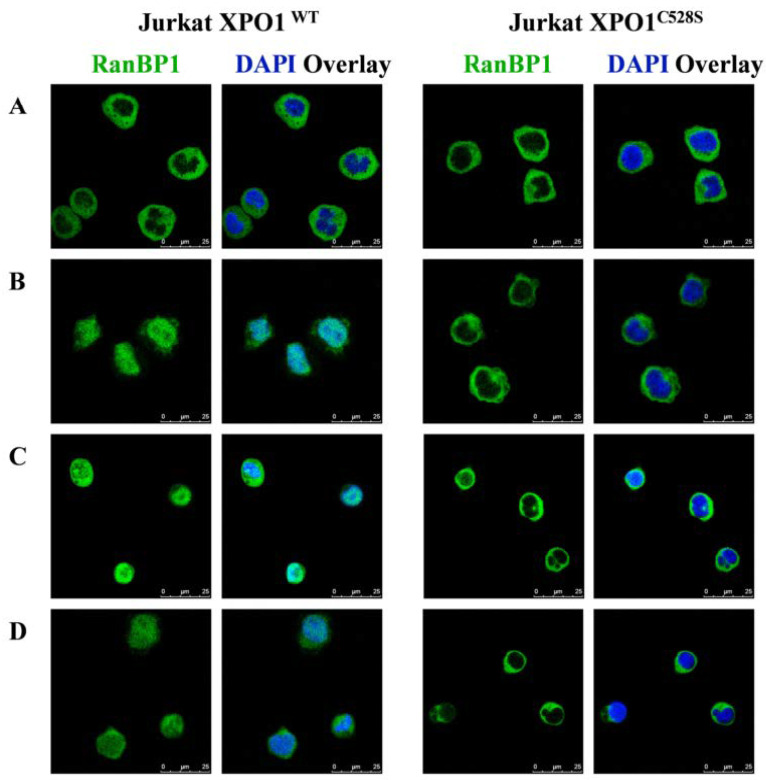
XPO1-mediated nuclear export of RanBP1 cargo protein. (**A**) Endogenous RanBP1 cargo protein (green) localized in the cytoplasm of untreated wild-type and mutant XPO1^C528S^ Jurkat cells; (**B**) 3 h after treatment with 1 µM KPT-330 (Selinexor), RanBP1 accumulates in the nucleus of wild-type cells (left) but remains localized in the cytoplasm of mutant XPO1^C528S^ cells (right); (**C**,**D**) 3 h after treatment with 4 µM of prototype compounds **9** or **10**, respectively, nuclear accumulation of RanBP1 is found in wild-type Jurkat cells. In contrast, no nuclear retention of the cargo was induced in the mutant XPO1^C528S^ cells. Scale bar 25 µm, and nuclei were counterstained with DAPI (blue).

Once XPO1 inhibition was confirmed, an additional experiment was performed for compounds **9** and **10** employing a Jurkat leukemia cell line where the cysteine residue at position 528 of XPO1 had been replaced by a serine residue (Jurkat XPO1^C528S^) [19,43]. The effect of the mutation on the activity of the inhibitors was assessed by the visualization of the subcellular localization of the XPO1 cargo RanBP1. As shown in Figure 4A (control experiment), in both wild-type and mutant Jurkat cells, RanBP1 is localized in the cytoplasm as a consequence of the correct nuclear export mediated by XPO1. When both cell lines were treated with KPT-330 at 1 μM (used as a positive control, Figure 4B), in the wild-type cell line, RanBP1 was accumulated in the nucleus, since KPT-330 blocked its binding to XPO1, whereas in the mutant cell line, KPT-330 was unable to interact with XPO1; therefore, RanBP1 remained in the cytoplasm. Similarly, when both cell lines were treated with our prototype compounds **9** and **10** at 4 μM (Figure 4C, D, respectively), RanBP1 accumulated in the nucleus in the case of the wild-type cells, whereas its nuclear export was not inhibited in the mutant cell line.

The IC_50_ values of compounds **9** and **10** regarding their XPO1 inhibitory activity in WT and C528S Jurkat cell lines are shown in Table 3. Both compounds **9** and **10** inhibited nuclear export of RanBP1 cargo protein with IC_50_ values of 2.2 and 0.3 μM, respectively, in the WT Jurkat cell line. These results were in agreement with the IC_50_ values obtained for XPO1 inhibition in the HeLa reporter cell line (Table 2), where the chalcone **10** was the most potent of the two. What is most relevant is that both compounds lost their inhibitory activity against the Jurkat XPO1^C528S^ cell line, similar to the positive control KPT-330. Thus, these experiments evidenced the importance of Cys528 for the XPO1 inhibitory activity of these chalcones.

Thus, compounds **9** and **10**, able to react with GSH in incubation studies, were among the most potent XPO1 inhibitors and also showed good antiproliferative activity. On the other hand, the α-CH_3_ chalcone **18**, which poorly reacted with GSH in the incubation assays, was unable to inhibit XPO1 and also did not show antiproliferative activity. The most puzzling data comes from the esters **13** and **30**, which were almost inactive against XPO1 and in antiproliferation assays. However, in the incubation studies with GSH, compound **13** seems to be as reactive as the α-H chalcone **9**. Certainly, many factors can be involved in the lack of activity of this chalcone. A potential explanation may arise by taking into account that the XPO1 inhibition assays are performed in a cell culture, so that the presence of esterases might convert the ester into the corresponding carboxylic acid. If this is the case, the resulting α-COOH chalcone would be very poorly electrophilic based on the reactivity described for α-X-2´3,4,4´-tetramethylchalcones [27], and thus, its capacity to behave as a Michael acceptor against XPO1 would be seriously compromised.

### 2.4. Docking Studies

Docking molecular studies have been carried out for compounds **9** and **10** with CovDock [44,45], a computational tool developed by Schrödinger to perform covalent docking, using the coordinates of the S. cerevisiae XPO1 protein in its complex with KPT-8602 [20]. Using this tool, a covalent bond is created between the SH of Cys539 (Cys528 in human XPO1) in the NES-binding cleft and the β-position of the double bond of the chalcone.

**Figure 5:**
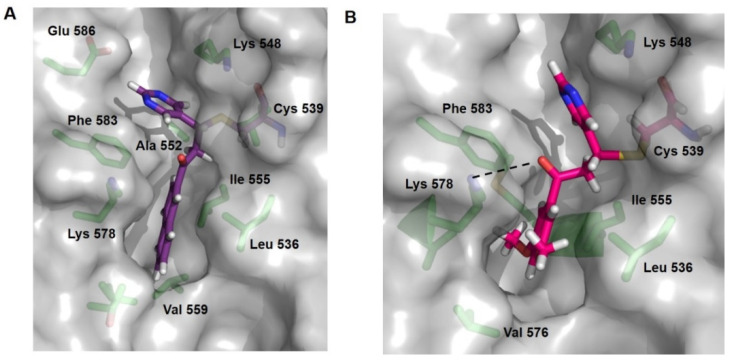
(**A**) Best CovDock solution of compound **9** (shown as purple sticks) at the NES-binding cleft of XPO1 (shown as light grey surface). (**B**) Best CovDock solution of compound **10** (shown as magenta sticks) at the NES-binding cleft of XPO1 (shown as light grey surface). Selected interacting residues from the NES-binding cleft are shown in green sticks and labeled, and hydrogen bond is shown as dashed lines.

The best-docked solution of compound **9** (Figure 5A) shows that the compound nicely fits within the upper part of the NES-binding cleft, so that the 2-naphthyl (ring A) has favorable interactions with the hydrophobic residues Ile555, Leu536, Phe583 and Val559, while the pyrimidin-5-yl ring (ring B) is buried in an inner region delimited by Ala552. As for compound **10** (Figure 5B), the best-docked solution indicates that the 3,5-dimethoxyphenyl ring lays in a hydrophobic cavity surrounded by the residues Ile555, Leu536 and Phe583, whereas ring B is directed to the upper part of the cleft. In addition, this binding mode is compatible with a hydrogen bond interaction between the carbonyl group of the ligand and the side chain of Lys579. This stabilizing interaction might help explain the higher inhibitory activity of compound **10** compared to **9** against XPO1. Additionally, this binding mode also suggests that one of the methoxy groups in ring A can be replaced by other longer ethers to gain additional interactions in the lower part of the cleft. Indeed, such ethers (compounds **33** and **34**) also showed XPO1 inhibition, but did not improve the inhibitory value of compound **10.**

## 3. Materials and Methods

### 3.1. Chemistry Procedures

Melting points were measured on a M170 apparatus (Mettler Toledo, Columbus, Ohio, USA) apparatus and are uncorrected. The elemental analysis was performed with a CHN-O-RAPID instrument (Heraus, Hanau, Germany). The elemental compositions of the compounds agreed within ±0.4% of the calculated values.

^1^H and ^13^C NMR spectra were recorded on a Varian INNOVA(now Agilent, Santa Clara, CA, USA) 300 operating at 299 MHz (^1^H) and 75 MHz (^13^C), respectively, a Varian INNOVA-400 operating at 399 MHZ (^1^H) and 99 MHz (^13^C), respectively, and a VARIAN SYSTEM-500 operating at 499 MHz (^1^H) and 125 MHz (^13^C), respectively. Monodimensional ^1^H and ^13^C spectra were obtained using standard conditions.

Compounds were also analyzed by HPLC/MS with a e2695 LC(Waters, Milford, Massachusetts, USA), coupled to a Waters 2996 photodiode array detector and a Waters Micromass ZQ. The column used is a Waters SunFire C18 2.1 × 50 mm, 3.5 µm, and the mobile phases were A: acetonitrile and B: H_2_O, together with a constant 5% of C (H_2_O with 2% formic acid) to assure 0.1% of formic acid along the run.

Analytical TLC was performed on silica gel 60 F_254_ (Merck, Dramstand, Germany)-precoated plates (0.2 mm). Spots were detected under UV light (254 nm) and/or charring with ninhydrin or phosphomolibdic acid.

Separations on silica gel were performed by preparative centrifugal circular thin-layer chromatography (CCTLC) on a Chromatotron^R^ (Kiesegel 60 PF_254_ gipshaltig (Merck)), with a layer thickness of 1 and 2 mm and a flow rate of 4 or 8 mL/min, respectively.

General procedure for the reaction of aromatic ethanones with aldehydes under basic conditions (General procedure A) [46].

To a solution of Ba(OH)_2_·8H_2_O (1.0–1.2 mmol) in water (0.2 mL), the corresponding aldehyde (1.0–2.0 mmol) in methanol (1 mL) was added. To the resultant mixture, the appropriate aromatic ketone (1.0–1.2 mmol) in methanol (8 mL) was added dropwise over 10 min and the reaction was stirred at room temperature for 2–16 h. The workup and purification procedures are described individually.

General procedure for the reaction of aromatic ketones with pyrimidine-5-carboxaldehyde under acid conditions (General procedure B) [47].

To a mixture containing the corresponding aromatic ketone (1.0 mmol) and pyrimidine-5-carboxaldehyde (1.2–2.8 mmol) in glacial acetic acid (1 mL), piperidine (0.5 mmol) was added dropwise, and then the reaction was stirred at 70–100 °C for 5–48 h. Volatiles were removed, and the residue was purified by CCTLC in the Chromatotron.

General procedure for the alkylation of phenol groups (General procedure C).

To a solution of the corresponding phenol (1.0 mmol) in anhydrous DMF (6 mL), Cs_2_CO_3_ (1.2–1.5 mmol) was added. After stirring at rt for 10 min, the appropriate alkyl halide (1.2 mmol) in anhydrous DMF (2 mL) was added dropwise. The resulting mixture was heated at 80 °C for 0.5–5 h and then quenched with water (5 mL). Volatiles were removed and the residue was diluted with ethyl acetate (20 mL) and washed with a saturated solution of NH_4_Cl (10 mL). The organic layer was dried over Na_2_SO_4_, filtered and evaporated to dryness. The residue was purified by CCTLC in the Chromatotron.

(E)-1-(Naphthalen-2-yl)-3-(pyrimidin-5-yl)prop-2-en-1-one (**9**)

Following the general procedure A, to a solution of Ba(OH)_2_·8H_2_O (111 mg, 0.35 mmol) and pyrimidine-5-carboxaldehyde (76 mg, 0.70 mmol) in a mixture of water (70 µL) and methanol (0.4 mL), 1-(naphthalen-2-yl)ethanone (**11**) (60 mg, 0.35 mmol) in methanol (2.8 mL) was added and the reaction was stirred for 2 h. The reaction was filtered, and the solid obtained was washed with cooled methanol and then purified by CCTLC (petroleum ether/ethyl acetate, 1:1) to yield 50 mg (55%) of **9** as a white solid. Mp: 175–177 °C. MS (ES, positive mode): m/z 261 (M+H)^+^. ^1^H NMR (300 MHz, DMSO-d_6_) δ: 7.61–7.77 (m, 2H, Ar), 7.81 (d, J = 15.8 Hz, 1H, CH=CH-Ar), 7.99–8.13 (m, 2H, Ar), 8.11–8.21 (m, 2H, Ar), 8.40 (d, J = 15.9 Hz, 1H, CH=CH-Ar), 8.99 (s, 1H, Ar), 9.23 (s, 1H, Ar), 9.37 (s, 2H, Ar). ^13^C NMR (75 MHz, DMSO-d_6_) δ: 124.4, 125.8, 127.5, 128.1, 129.0, 129.2, 129.3, 130.0, 131.3, 132.6, 134.7, 135.6, 137.3, 157.0, 159.3 (Ar, CH=CH), 188.9 (CO). Anal. calc. for (C_17_H_12_N_2_O): C, 78.44; H, 4.65; N, 10.76. Found: C, 78.19; H, 4.81; N, 10.53.

(E)-2-Methoxycarbonyl-1-(naphthalene-2-yl)-3-(pyrimidin-5-yl)prop-2-en-1-one (**13**)

Following general procedure B, a solution of methyl 3-(naphthalen-2-yl)-3-oxopropanoate (**12**) [28] (58 mg, 0.25 mmol), pyrimidine-5-carboxaldehyde (80 mg, 0.70 mmol) and piperidine (13 µL, 0.13 mmol) in glacial acetic acid (1 mL) was stirred at 100 °C overnight and evaporated. The residue was purified by CCTLC in the Chromatotron (DCM/ethyl acetate, 12:1) to yield 32 mg (40%) of **13** as a white solid. Mp: 161–163 °C. MS (ES, positive mode): m/z 319 (M+H)^+^. ^1^H NMR (400 MHz, DMSO-d_6_) δ: 3.76 (s, 3H, OCH_3_), 7.61 (ddd, J = 8.1, 6.8, 1.2 Hz, 1H, Ar), 7.70 (ddd, J = 8.2, 6.9, 1.3 Hz, 1H, Ar), 7.96–8.04 (m, 2H, Ar), 8.07 (d, J = 8.6 Hz, 1H, Ar), 8.08–8.15 (m, 2H, Ar, C=CH-Ar), 8.58 (d, J = 1.6 Hz, 1H, Ar), 8.77 (s, 2H, Ar), 9.04 (s, 1H, Ar). ^13^C NMR (101 MHz, DMSO-d_6_) δ: 53.4 (CH_3_), 123.7, 127.7, 127.8, 128.3, 129.7, 130.1, 130.4, 132.6, 132.7, 132.8, 134.9, 136.2, 136.6, 157.3, 159.1 (Ar, C=CH), 164.6, 194.5 (CO). Anal. calc. for (C_19_H_14_N_2_O_3_): C, 71.69; H, 4.43; N, 8.80. Found: C, 71.49; H, 4.53; N, 9.04.

3-(Naphthalen-2-yl)-3-oxopropanamide (**14**) 

The ester **12** (118 mg, 0.52 mmol) was dissolved in NH_3_/dioxane 0.5 N (2.5 mL) and heated at 110 °C for 7 h. Then, volatiles were removed, and the residue was redissolved in NH_3_/dioxane 0.5 N (2.5 mL) and heated at 110 °C overnight. Volatiles were removed, and the residue was purified by CCTLC in the Chromatotron (hexane/ethyl acetate, 1:2) to yield 37 mg (34%) of **14** as an amorphous solid containing a tautomeric mixture of the 3-oxopropanamide and 3-hydroxyacrylamide species (ratio 7:3). MS (ES, positive mode): m/z 214 (M+H)^+^. ^1^H NMR (400 MHz, DMSO-d_6_) δ: 4.00 (s, CH_2_), 5.89 (s, C(OH)=CH), 7.13 (br s, NH_2_), 7.38 (br s, NH_2_) 7.55–7.72 (m, Ar), 7.75 (dd, J = 8.7, 1.8 Hz, Ar), 7.94–8.07 (m, Ar), 8.09–8.14 (m, Ar), 8.33 (d, J = 1.9 Hz, Ar), 8.65–8.71 (m, Ar), 15.31 (s, C(OH)=CH).

(E)-2-Carbamoyl-1-(naphthalene-2-yl)-3-(pyrimidin-5-yl)prop-2-en-1-one (**15**) 

Following the general procedure B, a solution of **14** (33 mg, 0.16 mmol), pyrimidine-5-carboxaldehyde (20 mg, 0.19 mmol) and piperidine (10 µL, 0.10 mmol) in glacial acetic acid (1 mL) was stirred at 70 °C for 5 h and evaporated. The residue was purified by CCTLC in the Chromatotron (DCM/methanol, 75:1) to yield 17 mg (36%) of **15** as an amorphous solid. MS (ES, positive mode): m/z 304 (M+H)^+^. ^1^H NMR (500 MHz, DMSO-d_6_) δ: 7.61 (ddd, J = 8.2, 6.8, 1.2 Hz, 1H, Ar), 7.65 (br s, 1H, NH_2_), 7.69 (ddd, J = 8.2, 6.8, 1.3 Hz, 1H, Ar), 7.74 (s, 1H, C=CH-Ar), 7.93–7.96 (m, 2H, NH_2_, Ar), 7.99 (d, J = 8.2 Hz, 1H, Ar), 8.03 (d, J = 8.6 Hz, 1H, Ar), 8.08 (d, J = 8.2 Hz, 1H, Ar), 8.49 (s, 1H, Ar), 8.67 (s, 2H, Ar), 8.98 (s, 1H, Ar). ^13^C NMR (126 MHz, DMSO-d_6_): 124.0, 127.7, 128.2, 128.6, 129.4, 129.8, 130.2, 130.5, 132.2, 132.5, 133.5, 135.9, 140.0, 156.7, 158.5 (Ar, C=CH), 166.4, 195.8 (CO). Anal. calc. for (C_18_H_13_N_3_O_2_·0.5H_2_O): C, 69.22; H, 4.52; N, 13.45. Found: C, 68.95; H, 4.44; N, 13.26.

(E)-2-Methyl-1-(naphthalen-2-yl)-3-(pyrimidin-5-yl)prop-2-en-1-one (**18**) 

Naphthalene was reacted with propionyl chloride as described to provide 1-(naphthalen-2-yl)propan-1-one (**17**) [30] as the major product, together with a small proportion of its 1-isomer. MS (ES, positive mode): m/z 185 (M+H)^+^. This mixture (91 mg, 0.49 mmol), pyrimidine-5-carboxaldehyde (144 mg, 1.33 mmol) and piperidine (24 µL, 0.25 mmol) in glacial acetic acid (1 mL) was stirred at 100 °C for 48 h and evaporated, following the general procedure B. The residue was purified by CCTLC in the Chromatotron (DCM/ethyl acetate, 14:1) to yield 42 mg (30%) of **18** as an amorphous solid. MS (ES, positive mode): m/z 275 (M+H)^+^. ^1^H NMR (400 MHz, DMSO-d_6_) δ: 2.26 (d, J = 1.5 Hz, 3H, CH_3_), 7.17 (s, 1H, C=CH-Ar), 7.63 (ddd, J = 8.1, 6.8, 1.4 Hz, 1H, Ar), 7.68 (ddd, J = 8.2, 6.9, 1.5 Hz, 1H, Ar), 7.87 (dd, J = 8.5, 1.7 Hz, 1H, Ar), 8.03 (d, J = 8.0 Hz, 1H, Ar), 8.07 (d, J = 8.6 Hz, 1H, Ar), 8.13 (d, J = 8.1 Hz, 1H, Ar), 8.44 (s, 1H, Ar), 8.99 (s, 2H, Ar), 9.17 (s, 1H, Ar). ^13^C NMR (126 MHz, DMSO-d_6_) δ: 15.2 (CH_3_), 125.8, 127.4, 128.1, 128.8, 128.9, 129.9, 130.2, 131.5, 132.4, 133.7, 134.7, 135.1, 140.3, 157.5, 157.8 (Ar, C=CH), 198.2 (CO). Anal. calc. for (C_18_H_14_N_2_O): C, 78.81; H, 5.14; N, 10.21. Found: C, 78.39; H, 5.12; N, 9.89.

3-(Naphthalen-2-yl)-3-oxopropanenitrile (**20**) [32]

An Ace pressure tube was charged with a solution of methyl 2-naphthoate (**19**) (250 mg, 1.34 mmol) and acetonitrile (210 μL, 4.03 mmol) in anhydrous toluene (2.7 mL). Then, NaH (60% dispersion in mineral oil, 162 mg, 4.03 mmol) was added, and the resulting mixture was heated to 110 °C overnight. The reaction was allowed to warm to rt and quenched with water (5 mL). Then, it was diluted with DCM (15 mL) and washed with HCl 1N (10 mL) and brine. The organic layer was dried over Na_2_SO_4_, filtered and evaporated to dryness. The residue was purified by flash chromatography (DCM/methanol, 100:1) to yield 125 mg (48%) of **20** as an amorphous solid. MS (ES, positive mode): m/z 196 (M+H)^+^. ^1^H NMR (400 MHz, DMSO-d_6_) δ: 4.89 (s, 2H, CH_2_), 7.66 (ddd, J = 8.1, 6.8, 1.4 Hz, 1H, Ar), 7.72 (ddd, J = 8.2, 6.8, 1.4 Hz, 1H, Ar), 7.96 (dd, J = 8.6, 1.8 Hz, 1H, Ar), 8.03 (d, J = 8.0 Hz, 1H, Ar), 8.06 (d, J = 8.7 Hz, 1H, Ar), 8.12 (d, J = 7.9 Hz, 1H, Ar), 8.65 (m, 1H, Ar). ^1^H NMR data are similar to those previously described [32].

(E)-2-Cyano-1-(naphthalene-2-yl)-3-(pyrimidin-5-yl)prop-2-en-1-one (**21**) 

Following the general procedure B, a solution of **20** (30 mg, 0.15 mmol), pyrimidine-5-carbaldehyde (23 mg, 0.21 mmol) and piperidine (8 µL, 0.08 mmol) in glacial acetic acid (1 mL) was heated to 70 °C for 2 h and evaporated. After cooling down with an ice bath and adding cold ethanol (3 mL), a solid precipitated, which was filtered under vacuum and washed with cold ethanol to yield 16 mg (36%) of **21** as an amorphous yellow solid. MS (ES, positive mode): m/z 286 (M+H)^+^. ^1^H NMR (400 MHz, DMSO-d_6_) δ: 7.65–7.77 (m, 2H, Ar), 7.94 (dd, J = 8.5, 1.8 Hz, 1H, Ar), 8.07 (d, J = 8.1 Hz, 1H, Ar), 8.11–8.18 (m, 2H, Ar), 8.34 (s, 1H, C=CH-Ar), 8.66 (d, J = 1.8 Hz, 1H, Ar), 9.36 (s, 1H, Ar), 9.38 (s, 2H, Ar).

(E)-1-(Naphthalen-2-yl)-3-(pyridin-3-yl)prop-2-en-1-one (**24**) 

Following the general procedure A, to a solution of Ba(OH)_2_·8H_2_O (111 mg, 0.35 mmol) and 3-pyridinecarboxaldehyde (**21**) (66 μL, 0.70 mmol) in water (70 µL) and methanol (0.4 mL), 1-(naphthalen-2-yl)ethanone (**11**) (60 mg, 0.35 mmol) in methanol (2.8 mL) was added, and the reaction was stirred for 3 h. Volatiles were removed, and the residue was purified by CCTLC (hexane/ethyl acetate, 1:1) to yield 67 mg (73%) of **24** as a pale yellow solid. Mp: 123–125 °C. MS (ES, positive mode): m/z 260 (M+H)^+^. ^1^H NMR (300 MHz, DMSO-d_6_) δ: 7.53 (dd, J = 8.0, 4.8 Hz, 1H, Ar), 7.60–7.77 (m, 2H, Ar), 7.85 (d, J = 15.7 Hz, 1H, CH=CH-Ar), 7.97–8.13 (m, 2H, Ar), 8.10–8.22 (m, 2H, Ar), 8.28 (d, J = 15.7 Hz, 1H, CH=CH-Ar), 8.41 (dt, J = 8.1, 1.9 Hz, 1H, Ar), 8.64 (dd, J = 4.8, 1.6 Hz, 1H, Ar), 8.98 (s, 1H, Ar), 9.09 (d, J = 2.2 Hz, 1H, Ar). ^13^C NMR (75 MHz, DMSO-d_6_) δ: 124.3, 124.4, 127.4, 128.1, 128.9, 129.2, 130.0, 131.0, 131.1, 132.7, 135.0, 135.5, 135.6, 140.8, 150.7, 151.4 (Ar, CH=CH), 189.0 (C=O). Anal. calc. for (C_18_H_13_NO): C, 83.37; H, 5.05; N, 5.40. Found: C, 83.36; H, 5.23; N, 5.46. This compound has been recently described using slightly different reaction conditions [48].

(E)-1-(Naphthalen-2-yl)-3-(pyridin-2-yl)prop-2-en-1-one (**25**) 

Following the general procedure A, to a solution of Ba(OH)_2_·8H_2_O (111 mg, 0.35 mmol) and 2-pyridinecarboxaldehyde (**22**) (67 μL, 0.70 mmol) in water (70 µL) and methanol (0.4 mL), 1-(naphthalen-2-yl)ethanone (**11**) (60 mg, 0.35 mmol) in methanol (2.8 mL) was added, and the reaction was stirred for 7 h. Volatiles were removed, and the residue was purified by CCTLC (hexane/ethyl acetate, 6:1) to yield 37 mg (40%) of **25** as a pale yellow solid. Mp: 89–90 °C. MS (ES, positive mode): m/z 260 (M+H)^+^. ^1^H NMR (400 MHz, DMSO-d_6_) δ: 7.47 (ddd, J = 6.9, 4.7, 2.4 Hz, 1H, Ar), 7.62–7.73 (m, 2H, Ar), 7.80 (d, J = 15.4 Hz, 1H, CH=CH-Ar), 7.90–7.97 (m, 2H, Ar), 8.03 (d, J = 8.0 Hz, 1H, Ar), 8.04–8.15 (m, 2H, Ar), 8.23 (d, J = 7.9 Hz, 1H, Ar), 8.34 (d, J = 15.4 Hz, 1H, CH=CH-Ar), 8.72 (d, J = 4.5 Hz, 1H, Ar), 8.88 (d, J = 1.8 Hz, 1H, Ar). ^13^C NMR (101 MHz, DMSO-d_6_) δ: 124.4, 125.3, 125.6, 127.5, 128.2, 129.1, 129.3, 130.3, 131.0, 132.8, 135.1, 135.6, 137.7, 143.5, 150.5, 153.3 (Ar, CH=CH), 189.7 (CO). Anal. calc. for (C_18_H_13_NO): C, 83.37; H, 5.05; N, 5.40. Found: C, 83.22; H, 5.08; N, 5.26. This compound has been recently described using slightly different reaction conditions [48].

(E)-1-(Naphthalen-2-yl)-3-(pyridin-4-yl)prop-2-en-1-one (**26**) 

Following the general procedure A, to a solution of Ba(OH)_2_·8H_2_O (111 mg, 0.35 mmol) and 4-pyridinecarboxaldehyde (**23**) (52 μL, 0.53 mmol) in water (70 µL) and methanol (0.5 mL), 1-(naphthalen-2-yl)ethanone (**11**) (60 mg, 0.35 mmol) in methanol (2.8 mL) was added and the reaction was stirred for 2 h. Water was added, and the resulting solid was isolated and then purified by CCTLC (hexane/ethyl acetate/triethylamine, 3:1:0.04) to yield 30 mg (32%) of **26** as a pale yellow solid. Mp: 135–137 °C. MS (ES, positive mode): m/z 260 (M+H)^+^. ^1^H NMR (400 MHz, DMSO-d_6_) δ: 7.64–7.73 (m, 2H, Ar), 7.76 (d, J = 15.7 Hz, 1H, CH=CH-Ar), 7.87–7.92 (m, 2H, Ar), 8.01–8.10 (m, 2H, Ar), 8.12–8.20 (m, 2H, Ar), 8.35 (d, J = 15.7 Hz, 1H, CH=CH-Ar), 8.68–8.73 (m, 2H, Ar), 8.98 (s, 1H, Ar). ^13^C NMR (126 MHz, DMSO-d_6_) δ: 123.0, 124.5, 126.8, 127.5, 128.2, 129.1, 129.4, 130.1, 131.4, 132.7, 134.8, 135.7, 141.4, 142.3, 150.8 (Ar, CH=CH), 189.3 (CO). Anal. calc. for (C_18_H_13_NO): C, 83.37; H, 5.05; N, 5.40. Found: C, 83.30; H, 5.00; N, 5.18. This compound has been recently described using slightly different reaction conditions [48].

(E)-1-(3′,5′-Dimethoxyphenyl)-3-(pyrimidin-5′’-yl)prop-2-en-1-one (**10**)

Following the general procedure A, to a solution of Ba(OH)_2_·8H_2_O (132 mg, 0.42 mmol) and pyrimidine-5-carbaldehyde (91 mg, 0.84 mmol) in water (84 µL) and methanol (0.5 mL), 1-(3,5-dimethoxyphenyl)ethan-1-one **(28)** [33] (76 mg, 0.42 mmol) in methanol (3.4 mL) was added, and the reaction was stirred overnight. The reaction was filtered and the solid obtained was washed with cooled methanol and then purified by CCTLC in the Chromatotron (hexane/ethyl acetate, 1:3) to yield 72 mg (64%) of **10** as a white solid. Mp: 184–185 °C. MS (ES, positive mode): m/z 271 (M+H)^+^. ^1^H NMR (400 MHz, DMSO-d_6_) δ: 3.85 (s, 6H, OCH_3_), 6.83 (t, J = 2.3 Hz, 1H, Ar), 7.31 (d, J = 2.3 Hz, 2H, Ar), 7.74 (d, J = 15.8 Hz, 1H, CH=CH-Ar), 8.17 (d, J = 15.8 Hz, 1H, CH=CH-Ar), 9.21 (s, 1H, Ar), 9.34 (s, 2H, Ar). ^13^C NMR (101 MHz, DMSO-d_6_) δ: 56.1 (OCH_3_), 105.9, 106.9, 125.8, 129.2, 137.8, 139.5, 157.2, 159.4, 161.2 (Ar, CH=CH), 188.7 (CO). Anal. calc. for (C_15_H_14_N_2_O_3_): C, 66.66; H, 5.22; N, 10.36. Found: C, 66.79; H, 5,24; N, 10,33.

(E)-2-Methoxycarbonyl-1-(3′,5′-dimethoxybenzoyl)-3-(pyrimidin-5′-yl)prop-2-en-1-one (**30**) 

Following the general procedure B, a solution of methyl 3-(3′,5′-dimethoxyphenyl)-3-oxopropanoate **(29)** [34] (50 mg, 0.21 mmol), pyrimidine-5-carbaldehyde (31 mg, 0.29 mmol) and piperidine (10 µL, 0.10 mmol) in acetic acid (1 mL) was stirred at 70 °C overnight and evaporated. The residue was purified by CCTLC in the Chromatotron (hexane/ethyl acetate, 1:1) to yield 27 mg (39%) of **30** as a pale yellow solid. Mp: 81–82 °C. MS (ES, positive mode): m/z 329 (M+H)^+^. ^1^H NMR (400 MHz, DMSO-d_6_) δ: 3.76 (s, 3H, COOCH_3_), 3.77 (s, 6H, OCH_3_), 6.83 (t, J = 2.3 Hz, 1H, Ar), 7.00 (d, J = 2.3 Hz, 2H, Ar), 8.00 (s, 1H, C=CH-Ar), 8.73 (s, 2H, Ar), 9.10 (s, 1H, Ar). ^13^C NMR (75 MHz, DMSO-d_6_) δ: 53.1 (COOCH_3_), 55.9 (OCH_3_), 106.9, 127.6, 134.4, 136.4, 137.1, 157.2, 159.1, 161.3 (Ar, CH=CH), 164.4 (COOCH_3_), 193.83 (CO). Anal. calc. for (C_17_H_16_N_2_O_5_): C, 62.19; H, 4.91; N, 8.53. Found: C, 62.56; H, 4.99; N, 8.20.

1-(3-Methoxy-5-(2-methoxyethoxy)phenyl)ethan-1-one (**31**) 

3,5-Dihydroxyacetophenone (**27**) was reacted with methyl iodide as described [49] to provide 1-(3-hydroxy-5-methoxyphenyl)ethan-1-one (MS (ES, positive mode): m/z 167 (M+H)^+^) together with 1-(3,5-dimethoxyphenyl)ethan-1-one **(28)**. The 3-hydroxy derivative (52 mg, 0.31 mmol) was reacted with Cs_2_CO_3_ (151 mg, 0.46 mmol) and 2-bromoehtyl methyl ether (34 µL, 0.37 mmol) in anhydrous DMF (0.9 mL) at 80 °C for 5 h, following the general procedure C. After workup, the residue was purified by CCTLC in the Chromatotron (DCM/ethyl acetate, 40:1) to yield 54 mg (77%) of **31** as a colourless oil. MS (ES, positive mode): m/z 225 (M+H)^+^. ^1^H NMR (400 MHz, DMSO-d_6_) δ: 2.55 (s, 3H, COCH_3_), 3.31 (s, 3H, OCH_3_), 3.63–3.68 (m, 2H, OCH_2_), 3.80 (s, 3H, OCH_3_), 4.11–4.17 (m, 2H, OCH_2_), 6.78 (t, J = 2.3 Hz, 1H, Ar), 7.06 (m, 1H, Ar), 7.08 (m, 1H, Ar).

1-(3-Methoxy-5-((3-methylbut-2-en-1-yl)oxy)phenyl)ethan-1-one (**32**) 

As described for the synthesis of **31**, 1-(3-hydroxy-5-methoxyphenyl)ethan-1-one (67 mg, 0.40 mmol) was reacted with Cs_2_CO_3_ (196 mg, 0.60 mmol) and 3,3-dimethylallylbromide (58 µL, 0.48 mmol) in anhydrous DMF (1.2 mL) at 80 °C for 1 h, following the general procedure C. After workup, the residue was purified by CCTLC in the Chromatotron (DCM/ethyl acetate, 80:1) to yield 71 mg (75%) of **32** as a colourless oil. MS (ES, positive mode): m/z 235 (M+H)^+^. ^1^H NMR (400 MHz, DMSO-d_6_) δ: 1.72 (s, 3H, CH_3_), 1.74 (s, 3H, CH_3_), 3.79 (s, 3H, OCH_3_), 4.58 (d, J = 6.8 Hz, 2H, OCH_2_CH), 5.42 (m, 1H, OCH_2_CH), 6.76 (t, J = 2.3 Hz, 1H, Ar), 7.04 (dd, J = 2.3, 1.4 Hz, 1H, Ar), 7.07 (dd, J = 2.3, 1.3 Hz, 1H, Ar).

(E)-1-(3′-Methoxy-5′-(2-methoxyethoxy)phenyl)-3-(pyrimidin-5′’-yl)prop-2-en-1-one (**33**) 

Following the general procedure A, to a solution of Ba(OH)_2_·8H_2_O (94 mg, 0.30 mmol) and pyrimidine-5-carbaldehyde (40 mg, 0.37 mmol) in water (60 µL) and methanol (0.3 mL), the ketone **31** (69 mg, 0.30 mmol) in methanol (2.4 mL) was added, and the reaction was stirred for 1.5 h. Volatiles were removed, and the residue was purified by CCTLC in the Chromatotron (DCM/MeOH, 80:1) to yield 29 mg (32%) of **33** as an amorphous white solid. MS (ES, positive mode): m/z 325 (M+H)^+^. ^1^H NMR (400 MHz, DMSO-d_6_) δ: 3.32 (s, 3H, OCH_3_), 3.67–3.71 (m, 2H, OCH_2_), 3.84 (s, 3H, OCH_3_), 4.17–4.22 (m, 2H, OCH_2_), 6.84 (d, J = 2.3 Hz, 1H, Ar), 7.29 (dd, J = 2.3, 1.3 Hz, 1H, Ar), 7.34 (dd, J = 2.3, 1.4 Hz, 1H, Ar), 7.74 (d, J = 15.8 Hz, 1H, CH=CH-Ar), 8.17 (d, J = 15.8 Hz, 1H, CH=CH-Ar), 9.20 (s, 1H, Ar), 9.34 (s, 2H, Ar). ^13^C NMR (101 MHz, DMSO-d_6_) δ: 56.1, 58.6, 67.8, 70.8 (CH_2_, CH_3_), 106.3, 106.8, 107.7, 125.8, 129.2, 137.8, 139.5, 157.2, 159.4, 160.5, 161.2 (Ar, CH=CH), 188.7 (CO). Anal. calc. for (C_17_H_18_N_2_O_4_): C, 64.96; H, 5.77; N, 8.91. Found: C, 64.59; H, 5.68; N, 8.78.

(E)-1-(3′-Methoxy-5′-((3-methylbut-2-en-1-yl)oxy)phenyl)-3-(pyrimidin-5′’-yl)prop-2-en-1-one (**34**) 

Following the general procedure A, to a solution of Ba(OH)_2_·8H_2_O (82 mg, 0.26 mmol) and pyrimidine-5-carbaldehyde (34 mg, 0.31 mmol) in water (52 µL) and methanol (0.26 mL), the ketone **32** (61 mg, 0.26 mmol) in methanol (2.1 mL) was added and the reaction was stirred for 2 h. Water was added, and the resulting solid was isolated and then purified by CCTLC in the Chromatotron (hexane/ethyl acetate, 1:1) to yield 39 mg (41%) of **34** as an amorphous white solid. MS (ES, positive mode): m/z 325 (M+H)^+^. ^1^H NMR (300 MHz, DMSO-d_6_) δ: 1.74 (s, 3H, CH_3_), 1.75 (s, 3H, CH_3_), 3.84 (s, 3H, OCH_3_), 4.62 (d, J = 6.8 Hz, 2H, OCH_2_CH), 5.42 (m, 1H, OCH_2_CH), 6.82 (t, J = 2.2 Hz, 1H, Ar), 7.28 (t, J =2.2 Hz, 1H, Ar), 7.32 (t, J = 2.3 Hz, 1H, Ar) 7.73 (d, J = 15.8 Hz, 1H, CH=CH-Ar), 8.16 (d, J = 15.8 Hz, 1H, CH=CH-Ar), 9.20 (s, 1H, Ar), 9.34 (s, 2H, Ar). ^13^C NMR (101 MHz, DMSO-d_6_) δ: 18.5, 25.9, 56.1, 65.2 (CH_2_, CH_3_), 106.5, 106.6, 107.9, 120.0, 125.80, 129.2, 137.7, 138.1, 139.4, 157.2, 159.3, 160.4, 161.2 (Ar, CH=CH, C(CH_3_)_2_=CH), 188.7 (CO). Anal. calc. for (C_19_H_20_N_2_O_3_): C, 70.35; H, 6.21; N, 8.64. Found: C, 70.21; H, 6.29; N, 8.35.

### 3.2. GSH Reactivity Assay

A 500 µM sample of the tested compound was incubated with 5 mM reduced L-glutathione for 24 h at 37 °C with a final volume of 200 μL. As a solvent system, 20 mM PBS buffer pH 7.4 with 2 mM EDTA:DMSO (1:1) was used [50]. To perform the assay, stock solutions of 20 mM of the chalcones (**9**, **10**, **13** and **18**) and 15 mM of reduced L-glutathione were freshly prepared for every experiment and then diluted properly to give the final electrophile:nucleophile ratio (1:10). A 5 mM solution of 5,5′-dithiobis(2-nitrobenzoic acid) (DTNB) was prepared in the same solvent system to quench the reaction. After different time points (10 min, 1 h, 4 h and 24 h), an aliquot of 20 μL of the incubation mixture was quenched by adding 20 μL of the DTNB stock solution, mixed thoroughly, and then analyzed by HPLC. HPLC analysis was performed in Agilent 1120 compact LC, column ACE 5 C18-300 (15 cm × 4.6 mm); UV detection was performed at λ = 254 nm. Solvents used were acetonitrile for bottle A and H_2_O (containing 0.05% TFA) for bottle B, and the flow rate was 1mL/min. The gradients used were: (A) incubations with chalcone **9**: from 10% to 80% of solvent A in 10 min; (B) incubations with chalcones **10** and **18**: from 10% to 100% of solvent A in 10 min. The new peaks were analysed by LC-MS. As controls, a 500 μM solution of the α,β-unsaturated compound was also analysed, and a 5 mM reduced L-glutathione solution was quenched by adding DNTB at the same time points. In general, after 6 h of incubation, no GSH was detected.

### 3.3. Biological Assays

#### 3.3.1. Cell Culture and Reference Compounds

Cancer cell lines HCT-116, NCI-H460, HL-60, K-562 and Z-138 were acquired from the American Type Culture Collection (ATCC, Manassas, VA, USA). The DND-41 cell line was purchased from the Deutsche Sammlung von Mikroorganismen und Zellkulturen (DSMZ Leibniz-Institut, Brunswick, Germany), and the HAP-1 cell line was ordered from Horizon Discovery (Horizon Discovery Group, Water Beach, UK). All cell lines were cultured as recommended by the suppliers. Culture media were purchased from Gibco Life Technologies and supplemented with 10% fetal bovine serum (HyClone, GE Healthcare Life Sciences, Chicago, Illinois, USA).

Stably transfected HeLa NLS_SV40_-AcGFP-NES_PKI_ were cultured as described in Vercruysse et al. [19] CRISPR/Cas9 genome editing of the Jurkat cell line was performed as in Neggers et al. [43] to generate a XPO1^C528S^ mutant cell line.

Reference inhibitor KPT-330 was purchased from Selleckchem, and stock solutions were prepared in DMSO.

#### 3.3.2. Cell Proliferation Assays

Adherent cell lines HCT-116, NCI-H460 and Hap-1 cells were seeded at a density between 500 and 1500 cells per well in 384-well tissue culture plates (Greiner). After overnight incubation, cells were treated with different concentrations of the test compounds. Suspension cell lines HL-60, K-562, Z-138 and DND-41 were seeded at densities ranging from 2500 to 5500 cells per well in 384-well culture plates containing the test compounds at the same concentration points. The plates were incubated and monitored at 37 °C for 72 h in an IncuCyte (Essen BioScience Inc., Sartorius; Göttingen, Germany for real-time imaging of cell proliferation. Brightfield images were taken every 3 h, with one field imaged per well under 10× magnification. Cell growth was then quantified based on the percent cellular confluence, as analysed by the IncuCyte image analysis software, and used to calculate IC_50_ values by logarithmic interpolation. Compounds were tested in two independent experiments and represented as mean ± SEM.

#### 3.3.3. XPO1 Phenotypic Reporter Assay

To study the XPO1-mediated nuclear export, stably transfected HeLa NLS_SV40_-AcGFP-NES_PKI_ reporter cells were seeded at 8000 cells per well in 96-well all clear tissue culture plates (TPP). After overnight incubation, cells were treated with different doses of compound or solvent (DMSO) for 2 h and then fixed and counterstained with DAPI. Fluorescence was read on an ArrayScan XTI High Content Reader (Thermo Fisher Scientific, Waltham, MA, USA). Nuclear and cytoplasmic compartments were segmented and their average pixel intensities in the green channel were quantitated employing the HCS Studio software. Genedata Screener software was used for dose-response curve fitting, and calculation of EC_50_ values was based on the percentage of cells having a predominant nuclear localisation (ratio of nuclear to cytoplasmic signal equal or above 1.4) of the reporter construct. Compounds were tested in two independent experiments and represented as mean ± SEM.

#### 3.3.4. Immunofluorescence Staining of RanBP1

RanBP1 immunofluorescence staining was performed on both wild type and mutant XPO1^C528S^ Jurkat cells treated with compound or solvent (DMSO) for 3 h. Cells were harvested at 400× *g*, washed in PBS and then transferred into an 8-well µ-Slide (Ibidi) pretreated with 0.1% (*w*/*v*) poly-L-lysine (Sigma). Cells were allowed to adhere to the slides and then subsequently fixed (4% PFA in PBS), washed and permeabilized (0.2% Triton X-100 in PBS). Further immunofluorescence staining was then performed according to standard procedures. Employed antibodies were rabbit anti-RanBP1 (ab97659, Abcam, Cambridge, UK) at a 1:500 dilution and secondary Alexa Fluor 488 goat anti-rabbit antibody (A11008, Invitrogen, ThermoFisher Scientific). Cell nuclei were counterstained with DAPI, and the samples were imaged by confocal microscopy on a Leica TCS SP5 confocal microscope (Leica Microsystems, Weitzlar, Germany), employing a HCX PL APO 63× (NA 1.2) water immersion objective. Subsequently, fluorescence was read on an ArrayScan XTI High Content Reader (Thermo Fisher Scientific, Waltham, MA, USA). Nuclear and cytoplasmic compartments were segmented, and their average pixel intensities in the green channel were quantitated similarly as for the XPO1 phenotypic reporter assay.

### 3.4. Computational Methods

The crystal structure of XPO1 complex with KPT-8602 was retrieved from the Protein Data Bank [51] (pdb id: 5JLJ [20]).

The Schrödinger Suite v2018-3 has been used for all the computational studies [52]. The 3D structure of all compounds used in the modelling studies were generated using the graphical interface Maestro, and these were then optimized using the tool Macromodel. For the docking studies, all ligands were prepared with LigPrep in Maestro, and the receptor protein was prepared with the Protein Preparation Wizard.

Covalent docking was performed with CovDock [44]. The KPT-8602-XPO1 X-Ray structure was used for all covalent docking studies. Cys539 was selected as the reactive residue and Michael addition was selected as the reaction type. A grid box of 10 Å was defined. CovDock was used with the default parameters published in reference [44], except for the number of final poses per ligand, which was set up to 10. MMGBSA analysis was also chosen. Results were visually inspected and analysed using the computer graphics program PyMOL [53].

## 4. Conclusions

Most of the described XPO1 inhibitors are α,β-unsaturated carbonyl compounds able to react with Cys528 at the NES-binding cleft of XPO1. Based on these examples, we synthesized two series of chalcones with a six-membered N-heterocycle as ring B and tested their XPO1 inhibitory activity. Most of the synthesized compounds inhibited XPO1 function in a reporter cell line, and this inhibition nicely correlated with their antiproliferative activity in cell culture assays, with compounds **9**, **10**, **24** and **34** as the most potent. Moreover, in a mutant Jurkat cell line where the Cys528 of XPO1 had been mutated to a Ser (Jurkat XPO1^C528S^), the capacity of the prototype compounds 9 and 10 to inhibit XPO1 mediated nuclear export of the cargo protein was abolished, indicating the importance of Cys at position 528 for the inhibitory activity of our compounds, as also demonstrated for KPT-330 [43]. Finally, the interaction of the chalcones **9** and **10** with the NES-binding cleft has been analyzed through covalent docking with CovDock. Thus, these chalcones may represent an alternative scaffold in the search for XPO1 inhibitors.

## Figures and Tables

**Table 1:** Antiproliferative activity of the synthesized chalcones against different tumor cell lines.

	IC_50_ (µM) ^a^						
Comp	Hap-1 ^b^	HCT-116 ^b^	NCI-H460 ^b^	DND-41 ^b^	HL-60 ^b^	K-562 ^b^	Z-138 ^b^
**9**	2.3 ± 0.2	2.4 ± 0.2	2.1 ± 0.1	2.1 ± 0.5	2.0 ± 0.5	1.8 ± 0.08	1.8 ± 0.05
**10**	2.1 ± 0.2	4.7 ± 1.7	3.5 ± 1.5	1.5 ± 0.2	2.2 ± 0.3	7.2 ± 1.9	0.5 ± 0.05
**13**	≥54.9	≥15.5	≥47.1	≥30.6	>100	>100	>100
**15**	2.1 ± 0.7	5.3 ± 3.1	2.6 ± 0.7	8.9 ± 0.9	5.5 ± 3.6	2.1 ± 0.1	5.1 ± 2.7
**18**	>100	>100	>100	39.1 ± 5.3	≥40.9	48.5 ± 2.4	>100
**24**	1.6 ± 0.1	3.1 ± 0.9	4.3 ± 2.2	1.8 ± 0.5	3.5 ± 2.1	2.2 ± 0.03	1.6 ± 0.1
**25**	3.4 ± 0.5	3.9 ± 1.5	51.6 ± 11.7	2.6 ± 0.4	1.8 ± 0.05	4.8 ± 2.8	2.3 ± 0.5
**26**	2.3 ± 0.3	1.9 ± 0.4	7.7 ± 1.1	1.9 ± 0.4	0.9 ± 0.2	4.0 ± 0.2	1.1 ± 0.4
**30**	≥32.0	≥22.2	17.3 ± 4.6	43.2 ± 6.4	23.4 ± 1.9	42.9 ± 0.1	47.6 ± 1.1
**33**	4.3 ± 2.5	34.2 ± 6.5	16.9 ± 4.5	3.7 ± 1.6	10.0 ± 0.9	37.5 ± 0.9	1.5 ± 0.2
**34**	10.0 ± 2.0	52.9 ± 3.6	34.5 ± 5.2	10.1 ± 0.0	40.2 ± 10.1	4.1 ± 1.9	4.6 ± 2.7
**KPT-330**	0.07 ± 0.03	0.10 ± 0.1	0.12 ± 0.08	0.05 ± 0.0	0.11 ± 0.1	0.09 ± 0.0	0.40 ± 0.4

^a^ IC_50_: concentration of compound at which 50% of cell proliferation is inhibited. Mean value of two independent experiments ±SEM. ^b^ Hap-1: chronic myeloid leukemia; HCT-116: colorectal carcinoma; NCI-H460: lung carcinoma; DND-41: acute lymphoblastic leukemia; HL-60: acute myeloid leukemia; K-562: chronic myeloid leukemia; Z-138: non-Hodgkin lymphoma.

**Table 2:** XPO1 inhibition.

Comp	IC_50_ (µM) ^a^
**9**	2.46 ± 0.16
**10**	0.55 ± 0.19
**13**	>100
**5**	9.18 ± 3.28
**18**	>100
**24**	1.27 ± 0.29
**25**	7.28 ± 1.34
**26**	10.74 ± 3.24
**30**	87.34 ± 4.91
**33**	12.35 ± 1.28
**34**	1.59 ± 0.36
**KPT-330**	0.05

^a^ IC_50_ (50% inhibitory concentration) is given as the mean ± SEM of two independent experiments.

**Table 3:** IC_50_ values of **9** and **10** in means of XPO1 inhibition in wild-type and XPO1^C528S^ mutant Jurkat cell lines.

Comp	IC_50_ (μM) ^a^	
	Jurkat XPO1^WT^	Jurkat XPO1^C528S^
**KPT-330**	0.07 ± 0.01	>5
**9**	2.2 ± 0.03	>5
**10**	0.3 ± 0.3	>5

^a^ IC_50_ (50% inhibitory concentration) is given as the mean ±SD of two independent experiments.

## Data Availability

Data is contained within the article or supplementary material.

## Supplementary Materials

The following are available online at https://www.mdpi.com/article/10.3390/ph14111131/s1, Figures S1–S4: Chromatograms of the incubations of chalcones **9**, **10**, **13** and **18** with GSH. ^1^H and ^13^C NMR spectra of the tested compounds.
