# Supplementary material for: Inhibition of XPO-1 Mediated Nuclear Export through the Michael-Acceptor Character of Chalcones
Source: Pharmaceuticals (Basel). 2021 Nov 6;14(11):1131. doi: 10.3390/ph14111131 (PMC8621101; doi:10.3390/ph14111131)

Includes:

- Chromatograms of the incubations of chalcones **9, 10, 13** and 1**8** with GSH (Figures S1-S4) S2
- ^{1}H and ^{13}C NMR spectra of the synthesized compounds S6

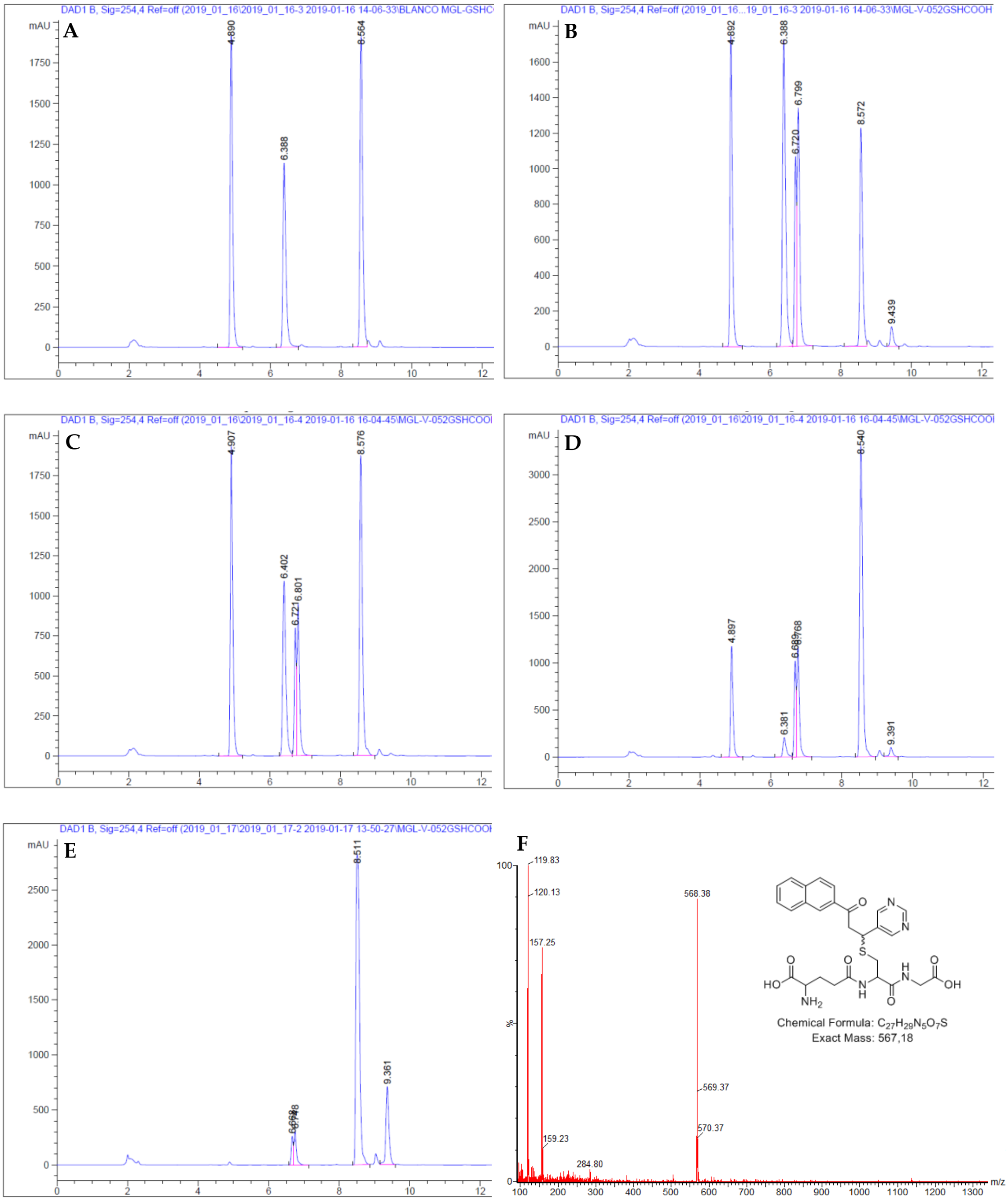

Figure S1. (A) Chromatogram of the incubation of GSH, in the absence of chalcone, quenched with DTNB (R_t = 8.5 min), giving the reaction adduct with GSH (R_t = 6.4 min) and TNB^{2-} (R_t = 4.9 min); (B-E) chromatograms of the incubation of chalcone **9** (R_t = 9.4 min) and GSH quenched with DTNB after 10 min (B), 1 h, (C), 4 h (D) and 24 h (E); (F) mass spectrum of the Michael addition products (R_t = 6.7, 6.8 min).

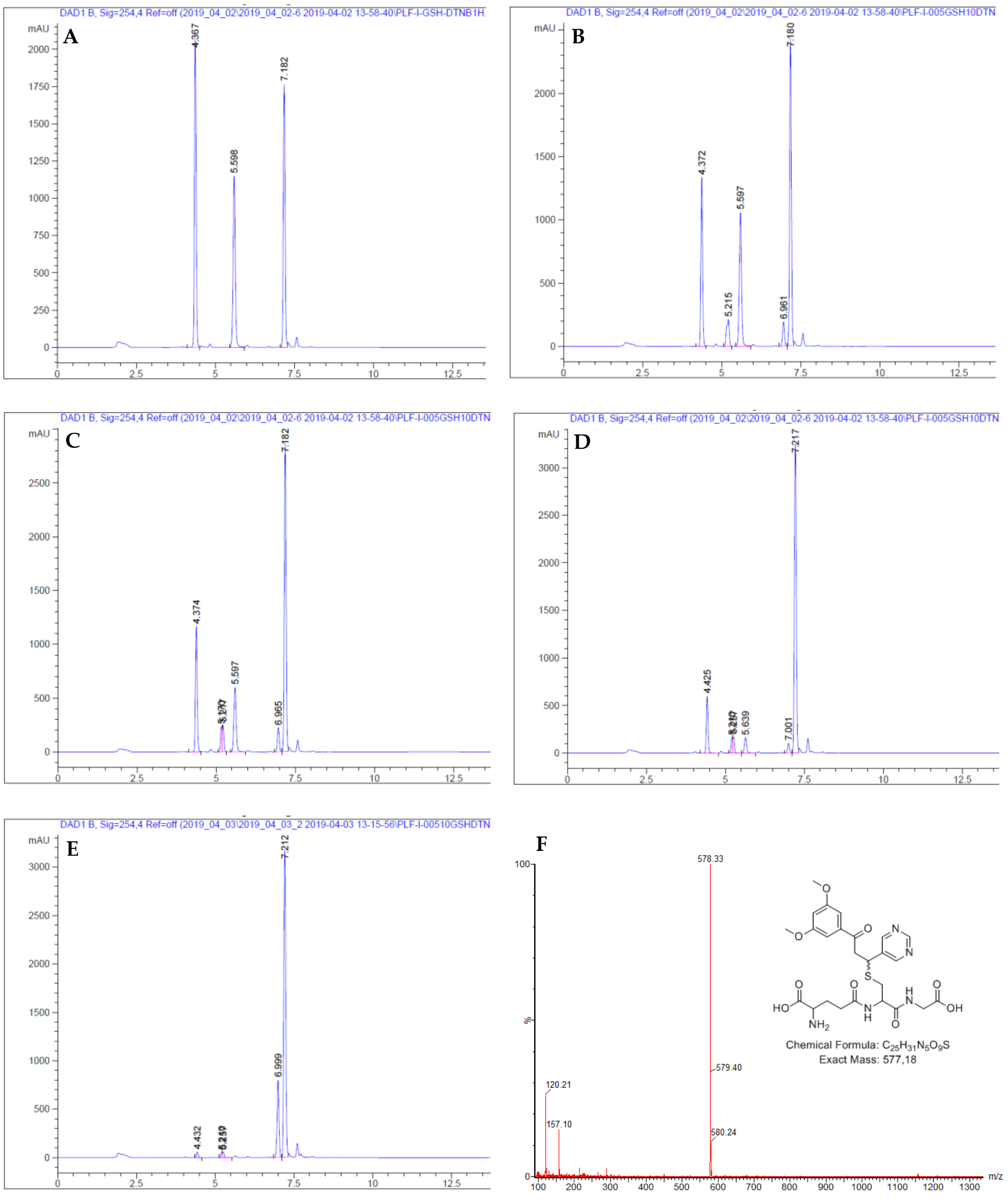

Figure S2. (A) Chromatogram of the incubation of GSH, in the absence of chalcone, quenched with DTNB (R_t = 7.2 min) giving the reaction adduct with GSH (R_t = 5.6 min) and TNB^{2-} (R_t = 4.4 min); (B-E) chromatograms of the incubation of chalcone **10** (R_t = 7.0 min) and GSH quenched with DTNB after 10 min (B), 1 h (C), 4 h (D) and 24 h (E); (F) mass spectrum of the Michael addition products (R_t = 5.21, 5.25 min).

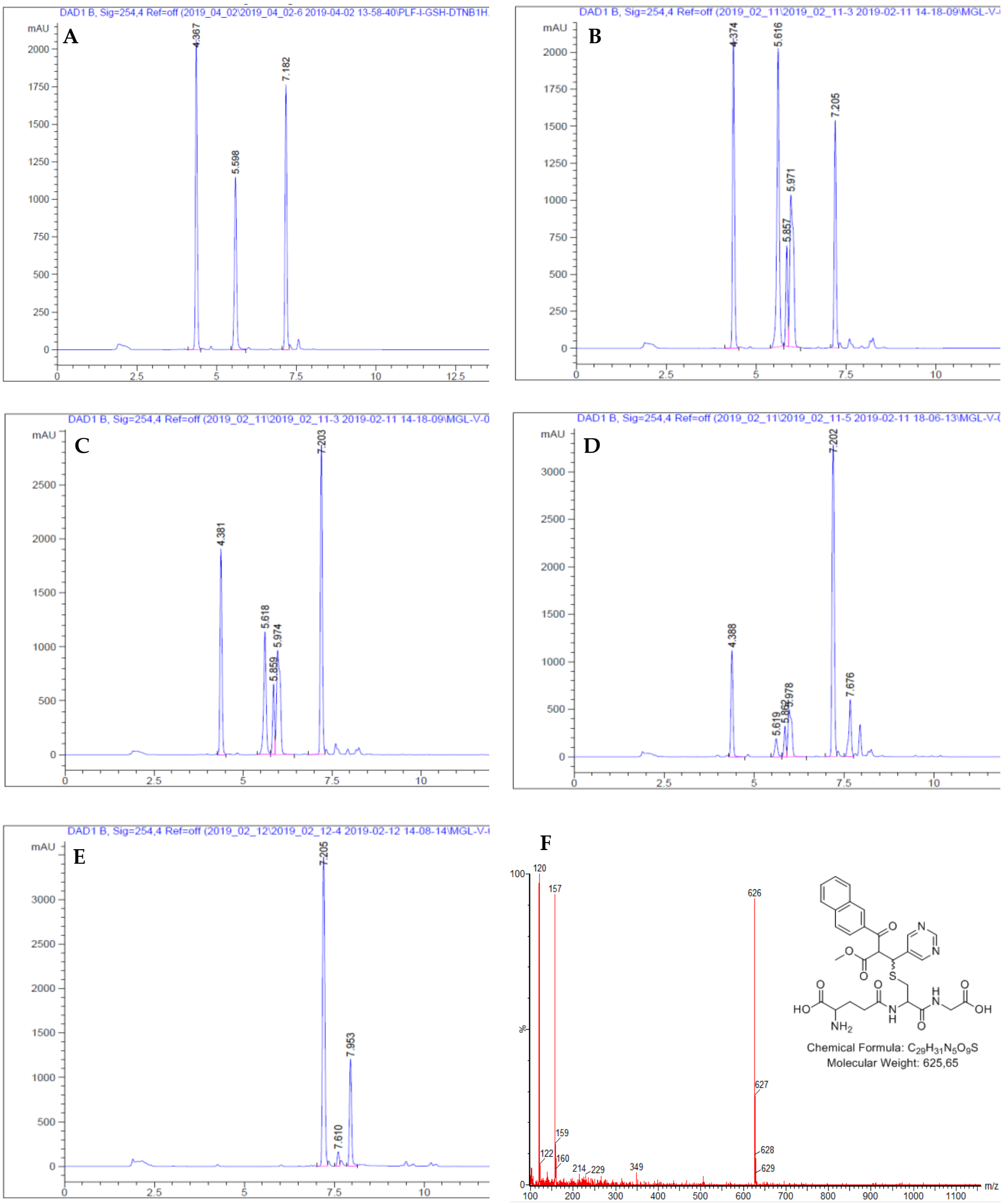

Figure S3. (A) Chromatogram of the incubation of GSH, in the absence of chalcone, quenched with DTNB (R_t = 7.2 min) giving the reaction adduct with GSH (R_t = 5.6 min) and TNB^{2-} (R_t = 4.4 min); (B-E) chromatograms of the incubation of chalcone **13** (R_t = 7.9 min) and GSH quenched with DTNB after 10 min (B), 1 h (C), 4 h (D) and 24 h (E); (F) mass spectrum of the Michael addition products (R_t = 5.86, 5.97 min).

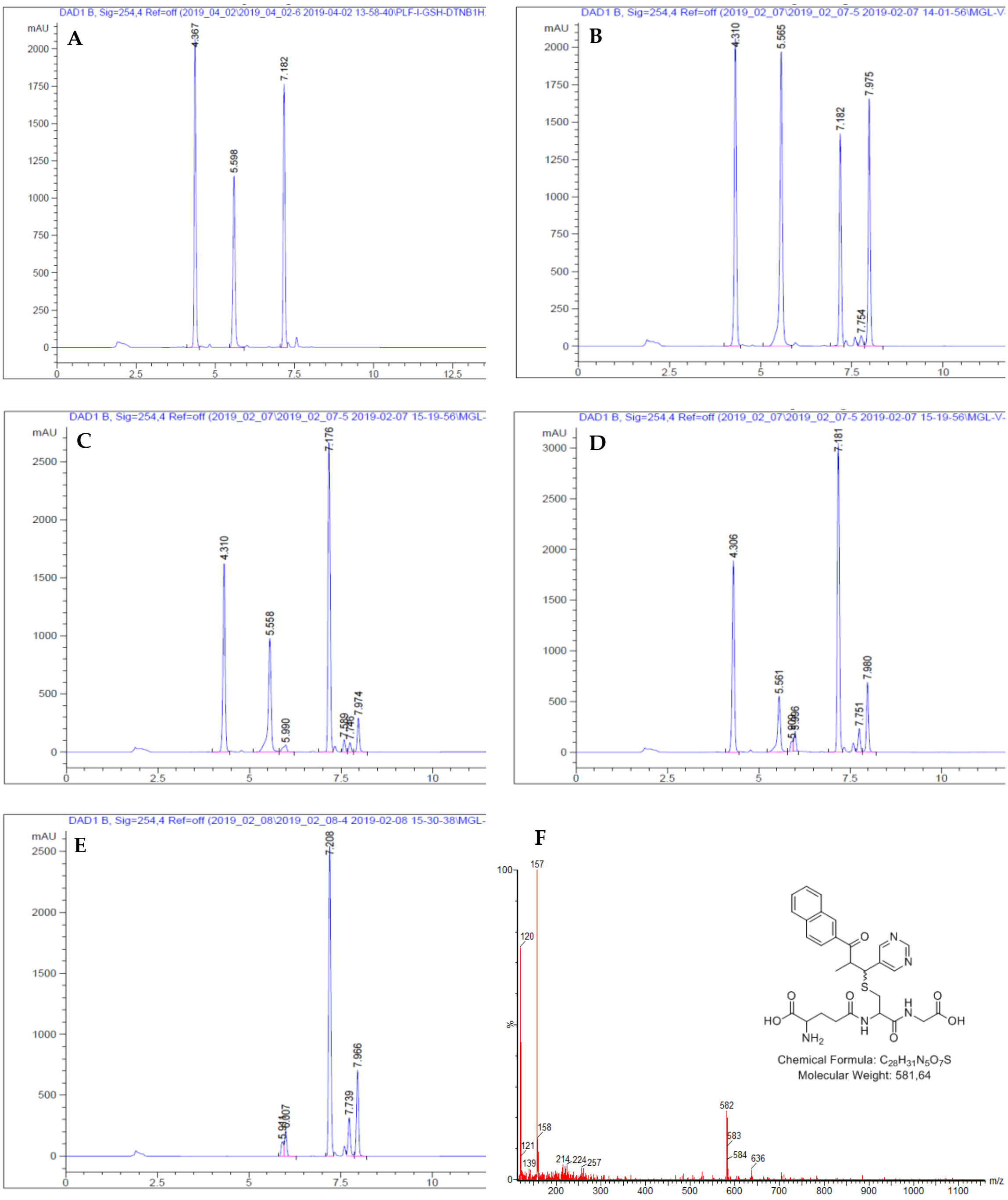

Figure S4. (A) Chromatogram of the incubation of GSH, in the absence of chalcone, quenched with DTNB (R_t = 7.2 min) giving the reaction adduct with GSH (R_t = 5.6 min) and TNB^{2-} (R_t = 4.3 min); (B-E) chromatograms of the incubation of chalcone **18** (R_t = 8.0 min) and GSH quenched with DTNB after 10 min (B), 1 h (C), 2 h (D) and 24 h (E); (F) mass spectrum of the Michael addition products (R_t = 5.98, 6.00 min).

Compound 9

^{1}H NMR (300 MHz, DMSO-d_6)

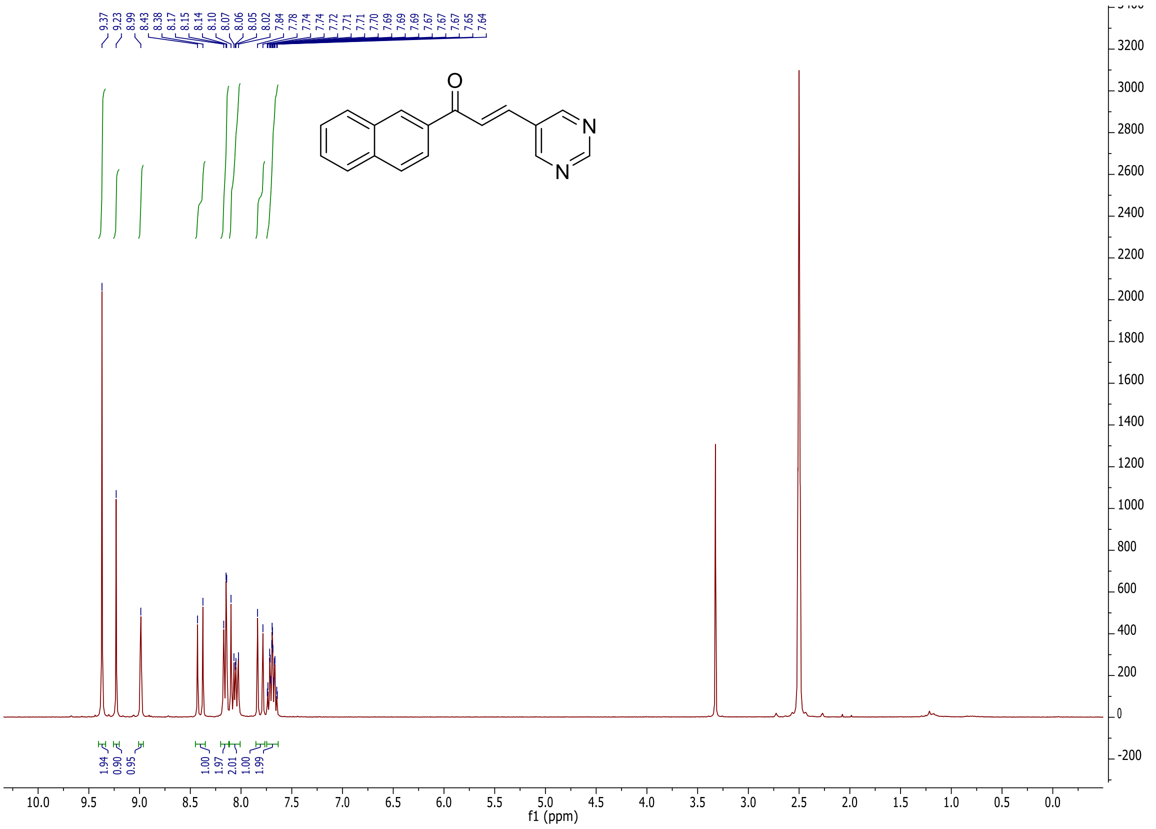

^{13}C NMR (75 MHz, DMSO-d_6)

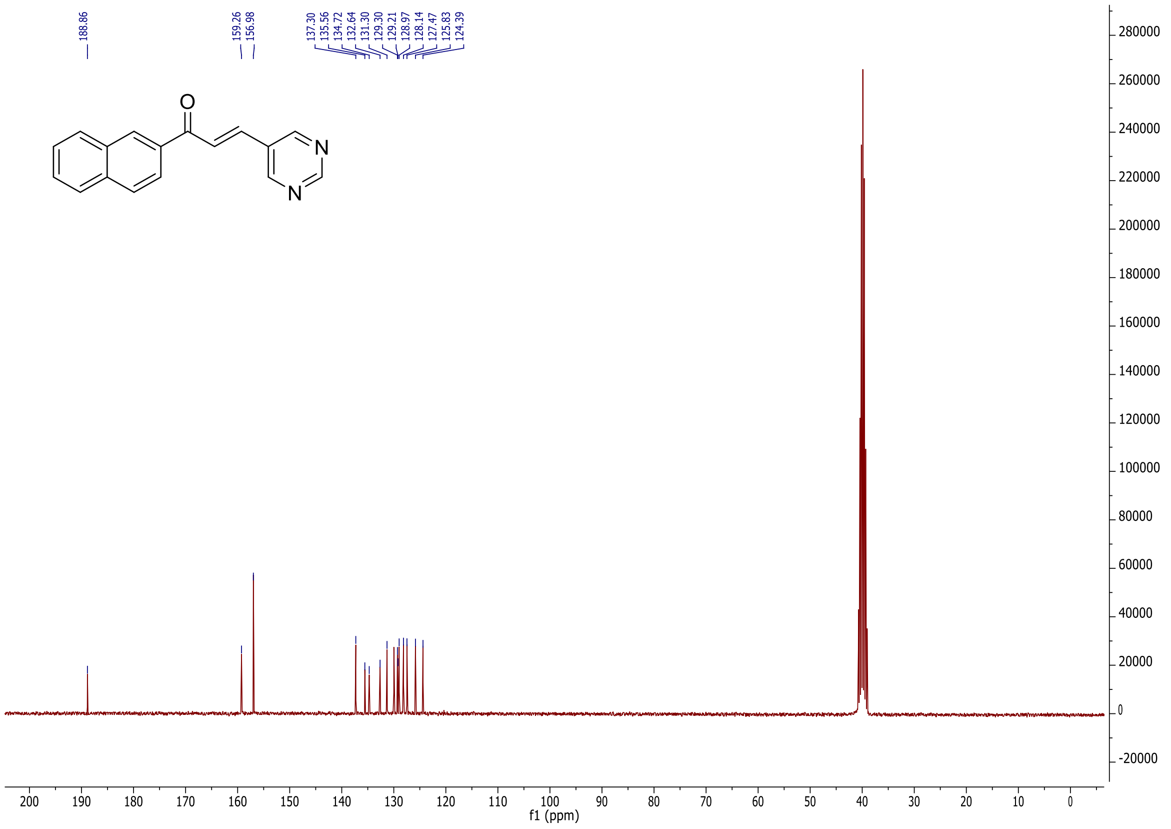

Compound 13

^{1}H-NMR (400 MHz, DMSO-d_6)

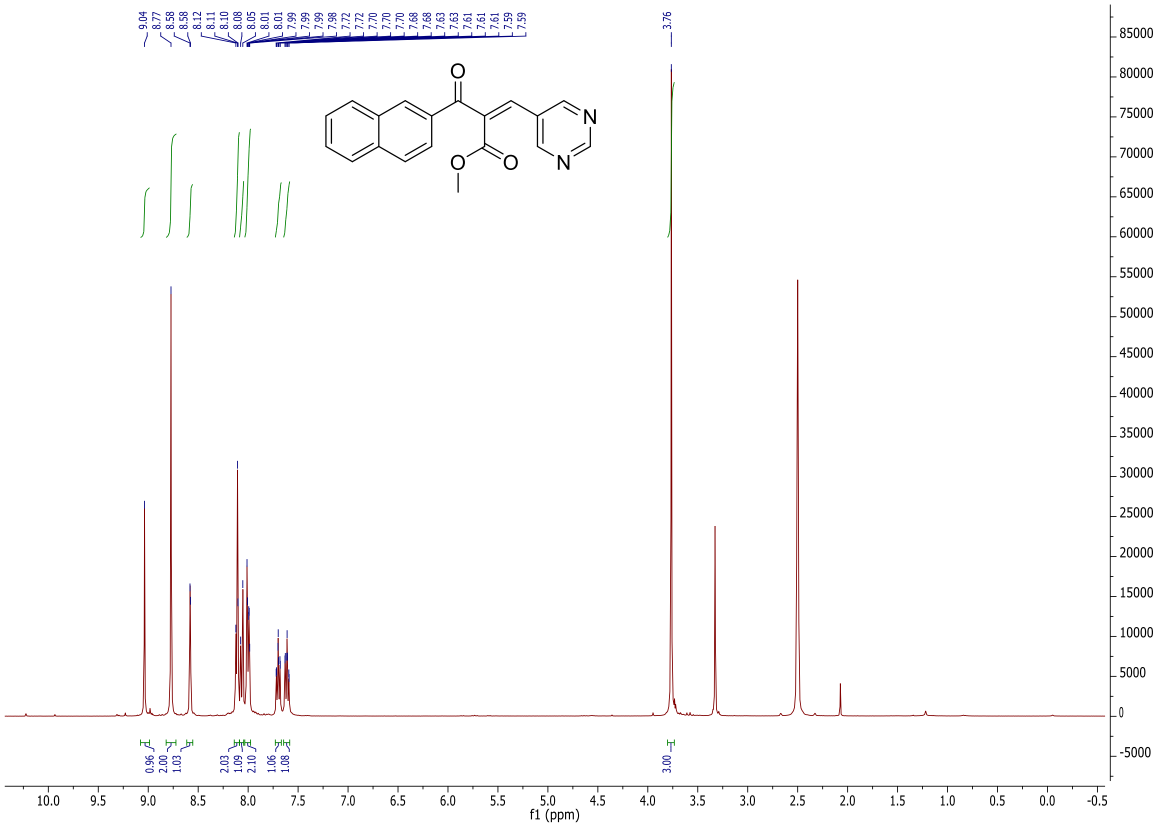

^{13}C-NMR (101 MHz, DMSO-d_6)

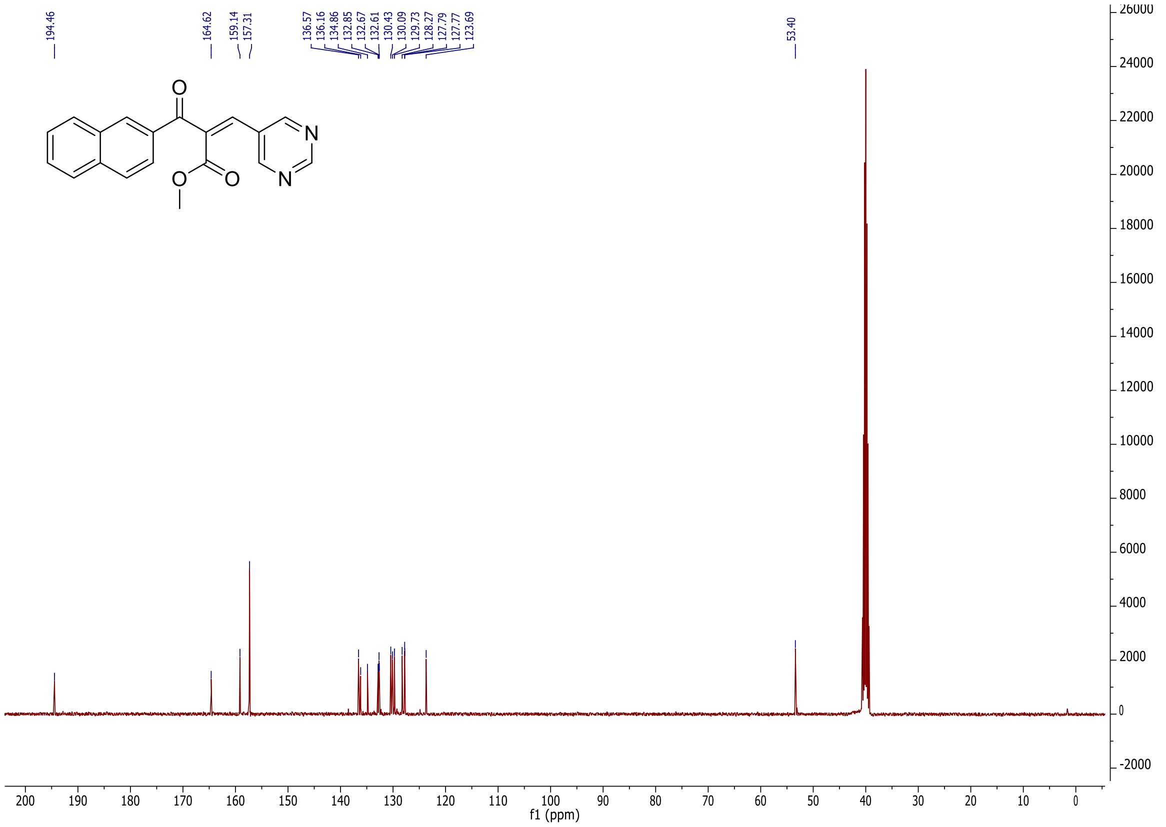

Compound 15

^{1}H-NMR (500 MHz, DMSO-d_6)

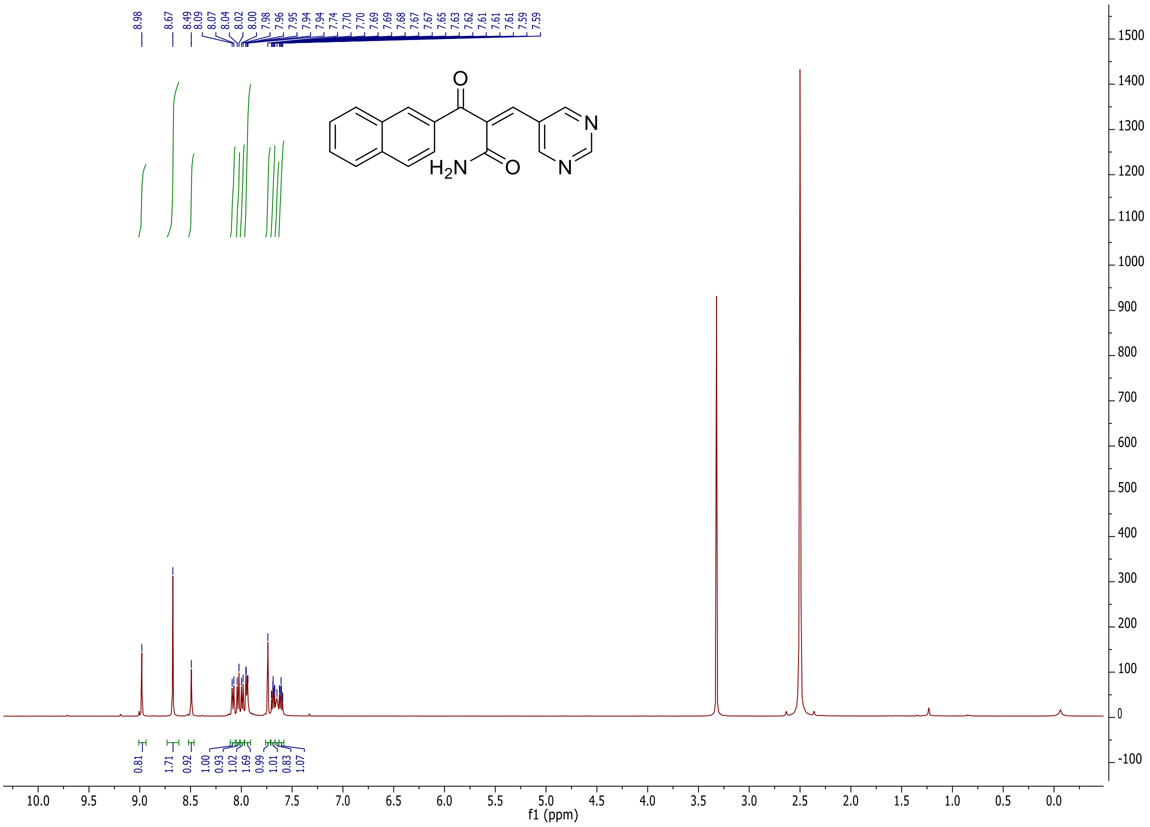

^{13}C-NMR (126 MHz, DMSO-d_6)

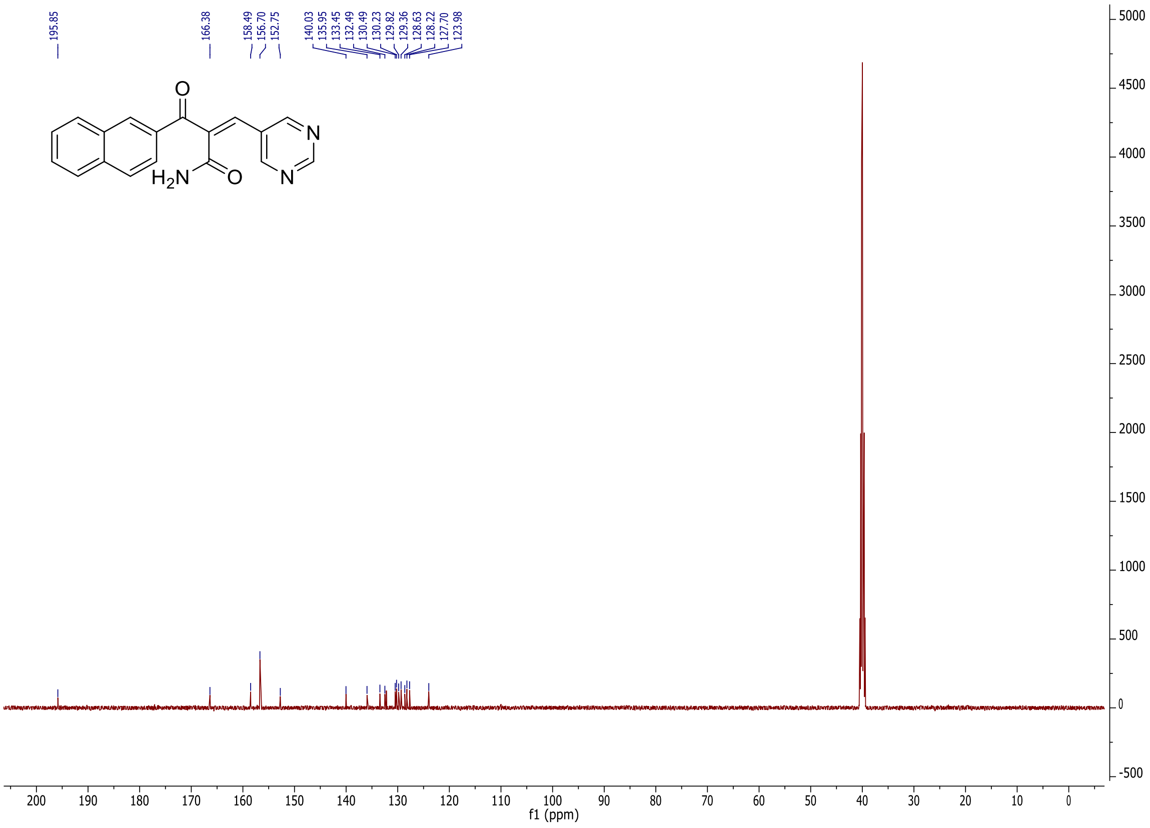

Compound 18

^{1}H-NMR (400 MHz, DMSO-d_6)

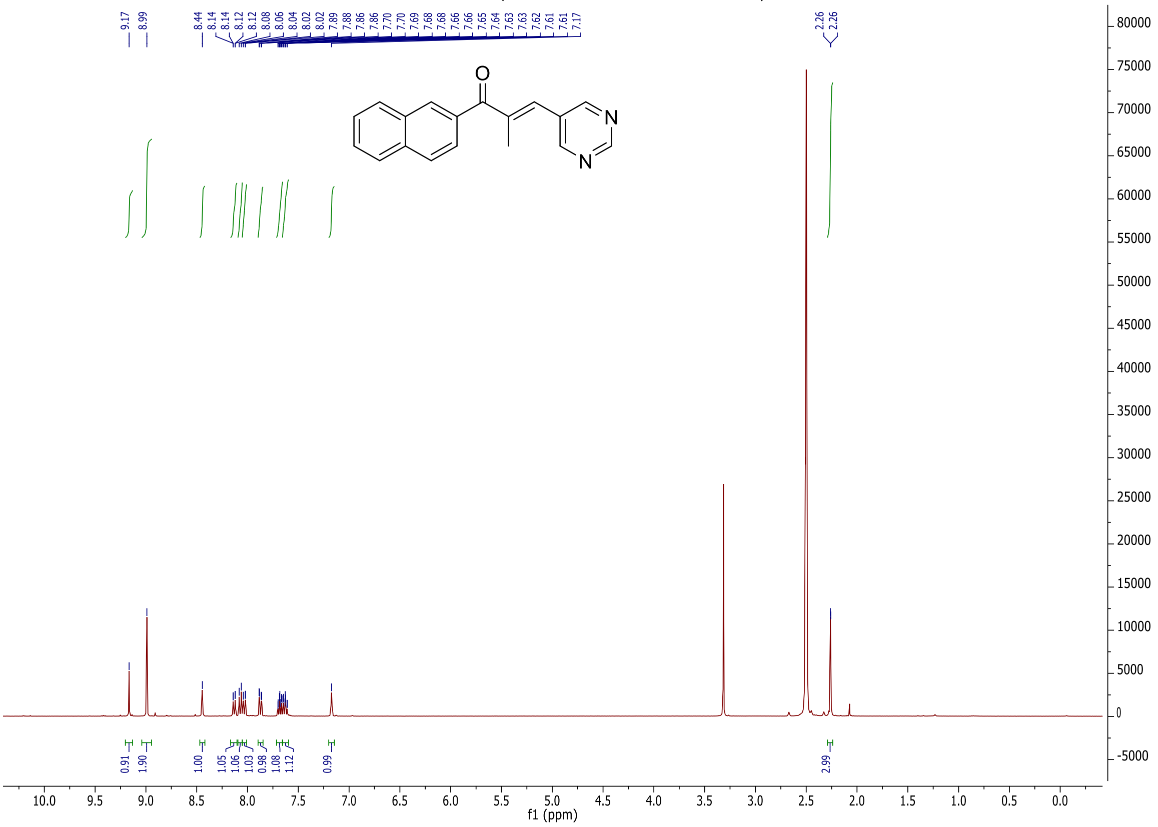

^{13}C-NMR (126 MHz, DMSO-d_6)

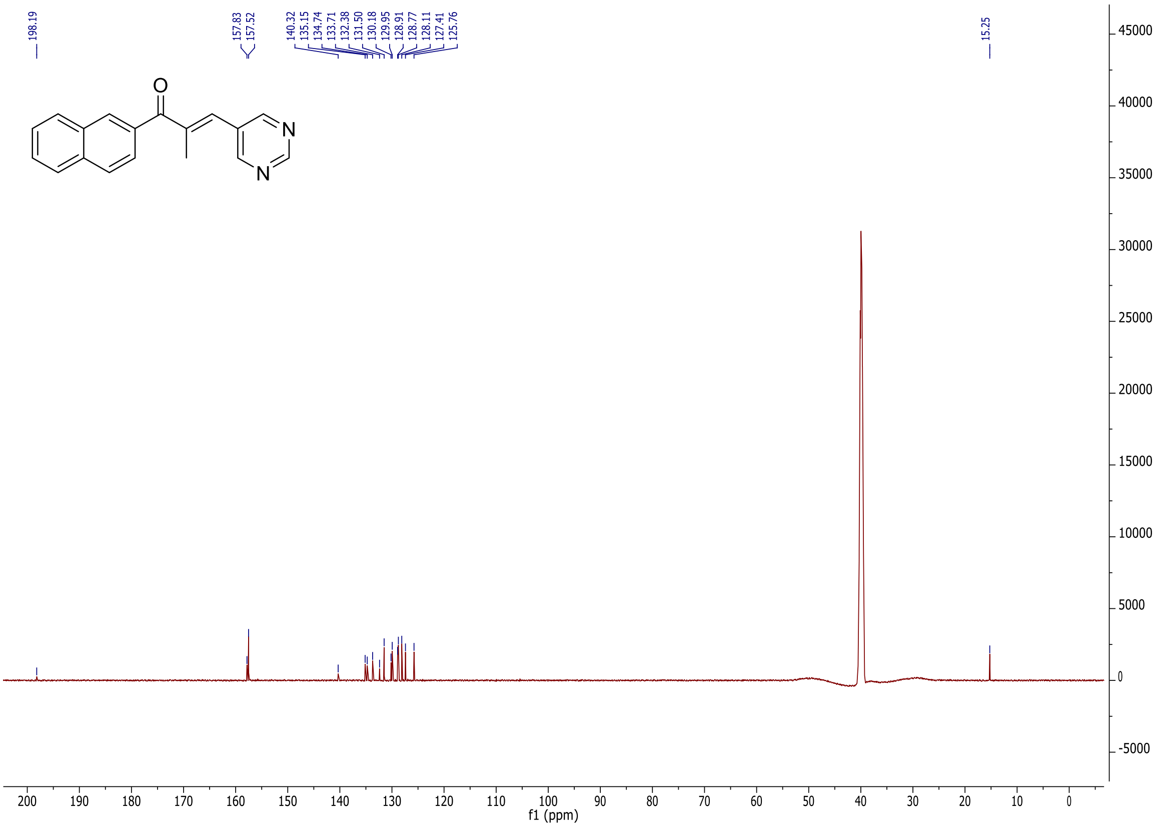

Compound 21

^{1}H-NMR (400 MHz, DMSO-d_6)

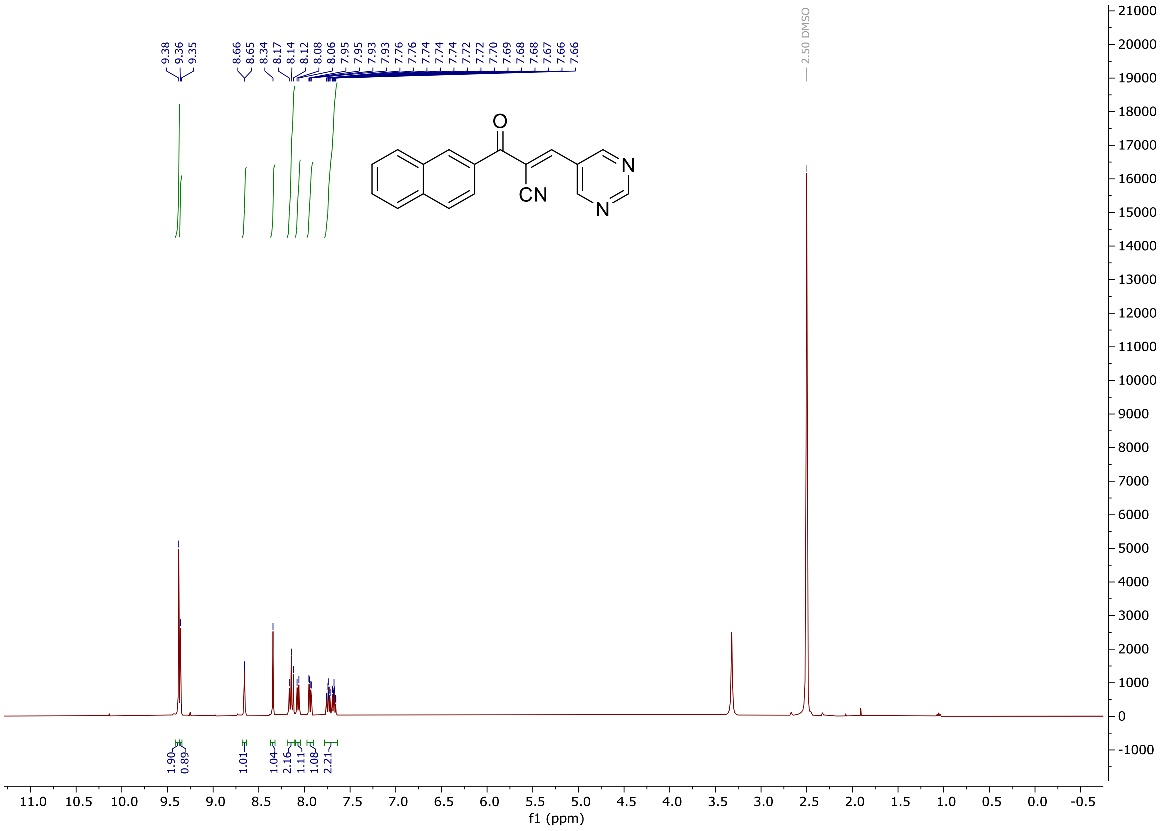

Compound 24

¹H-NMR (300 MHz, DMSO-d_6)

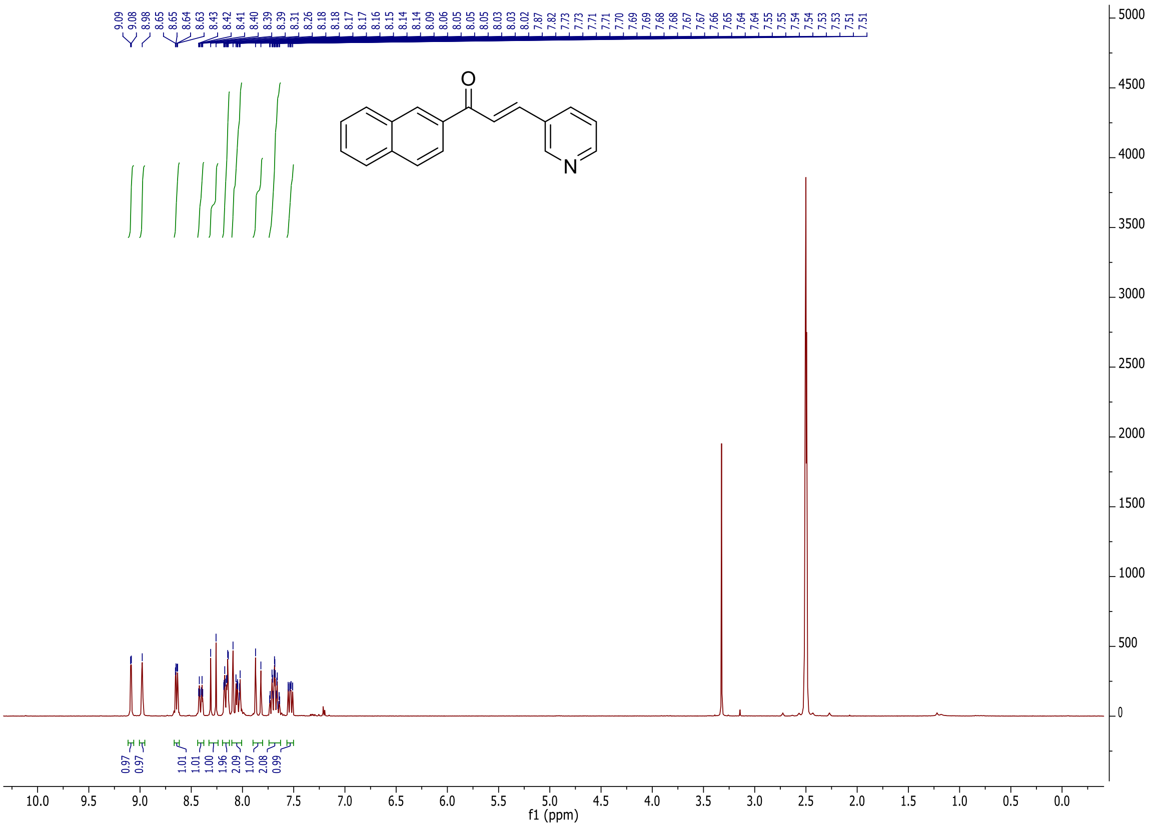

¹³C-NMR (75 MHz, DMSO-d_6)

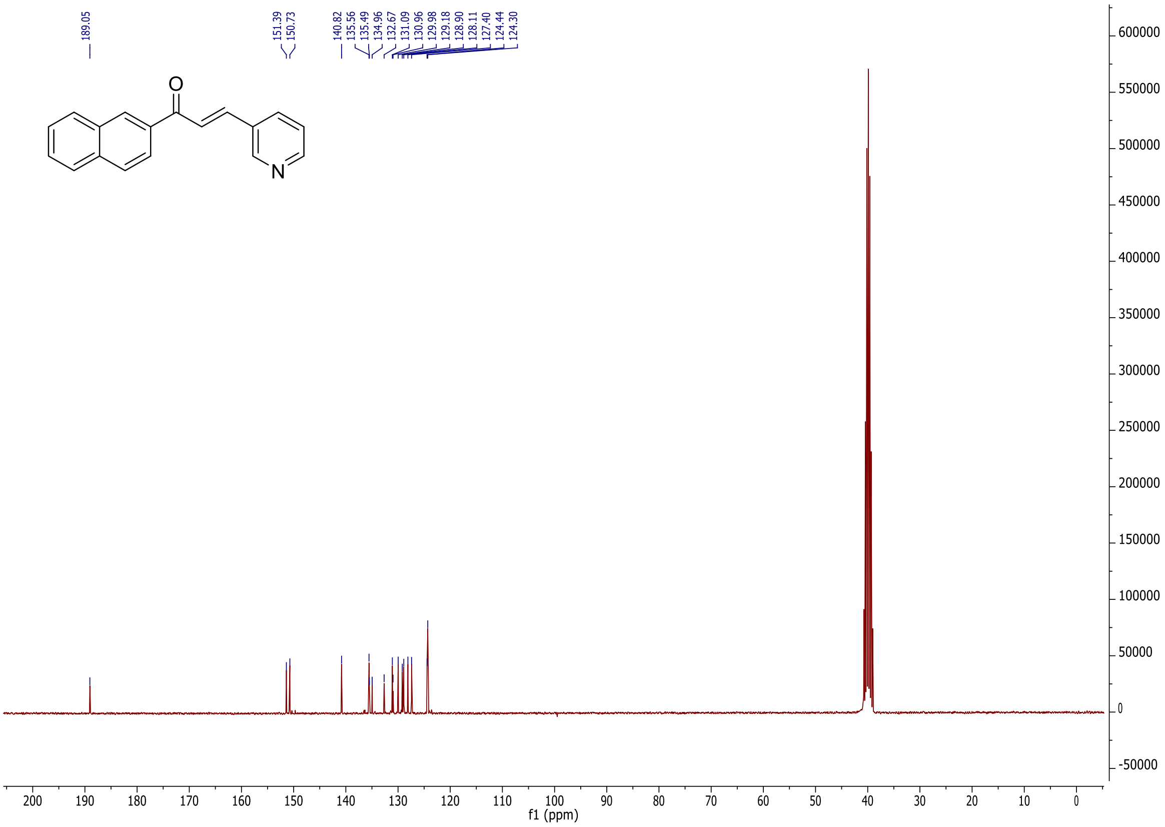

Compound 25

^{1}H-NMR (400 MHz, DMSO-d_6)

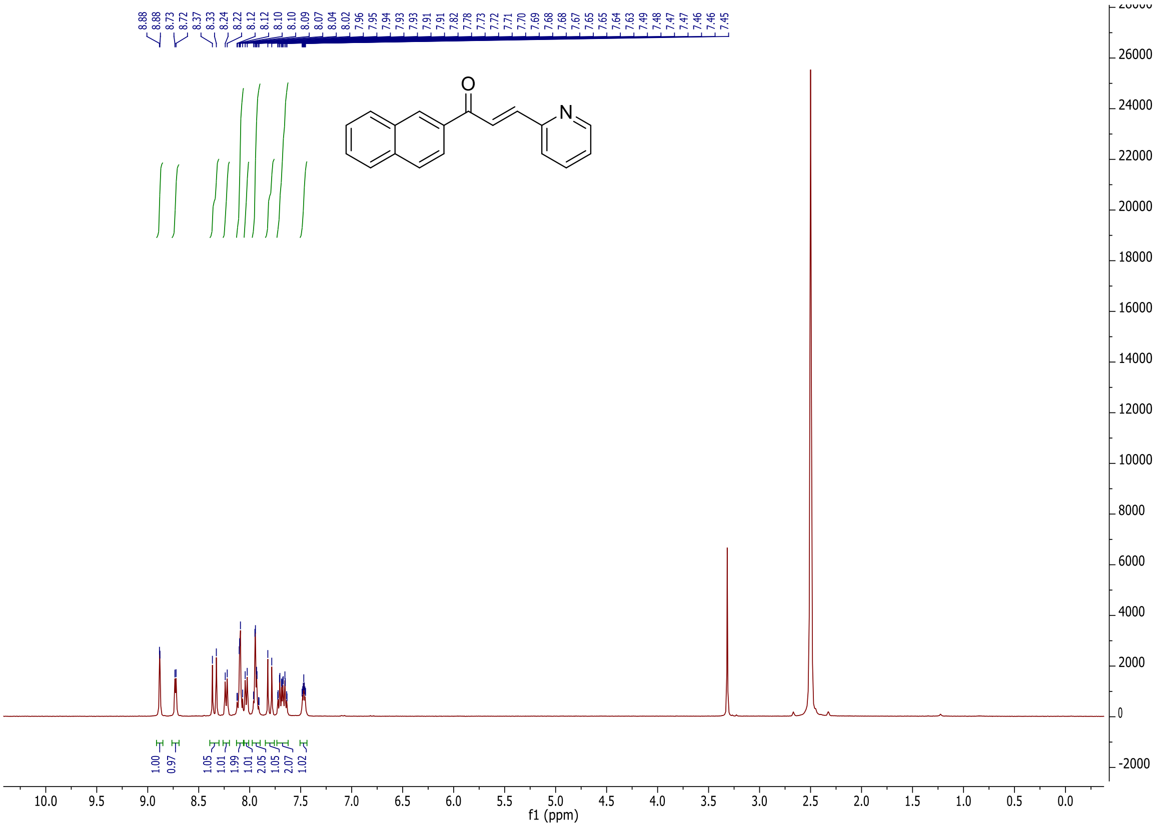

^{13}C-NMR (101 MHz, DMSO-d_6)

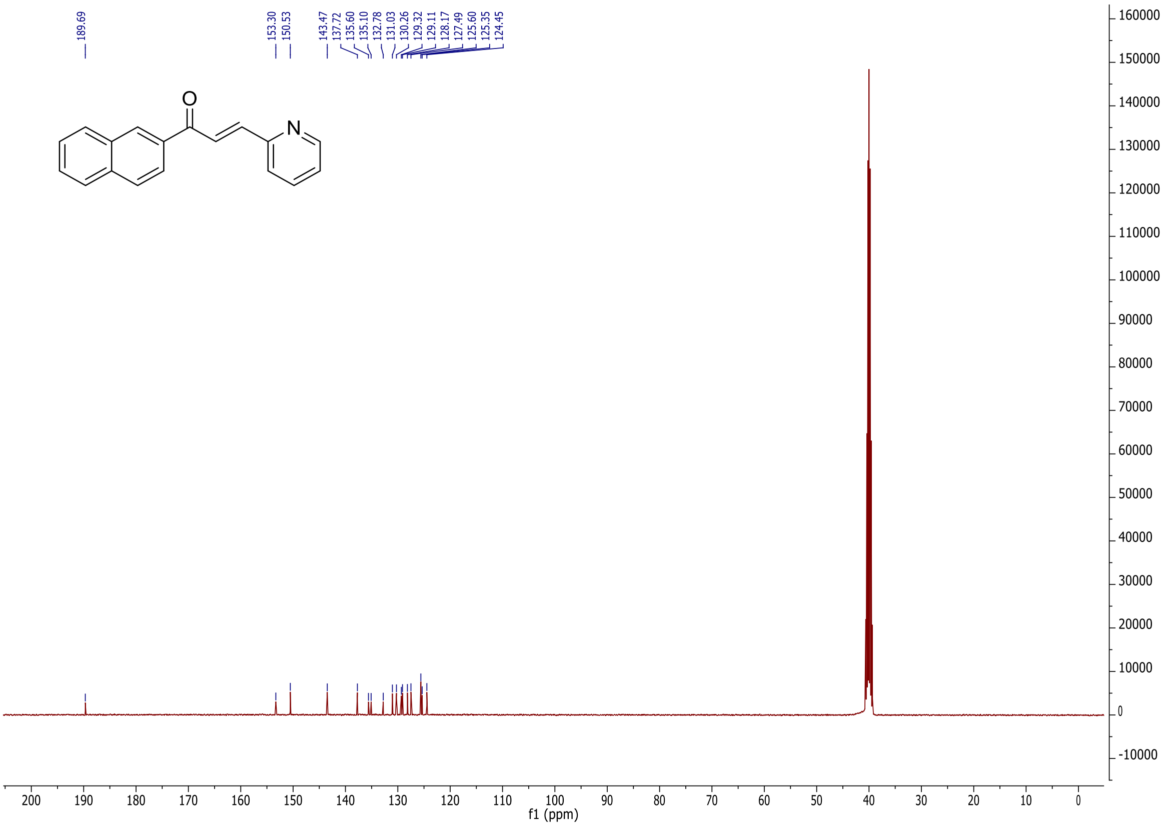

Compound 26

^{1}H-NMR (400 MHz, DMSO-d_6)

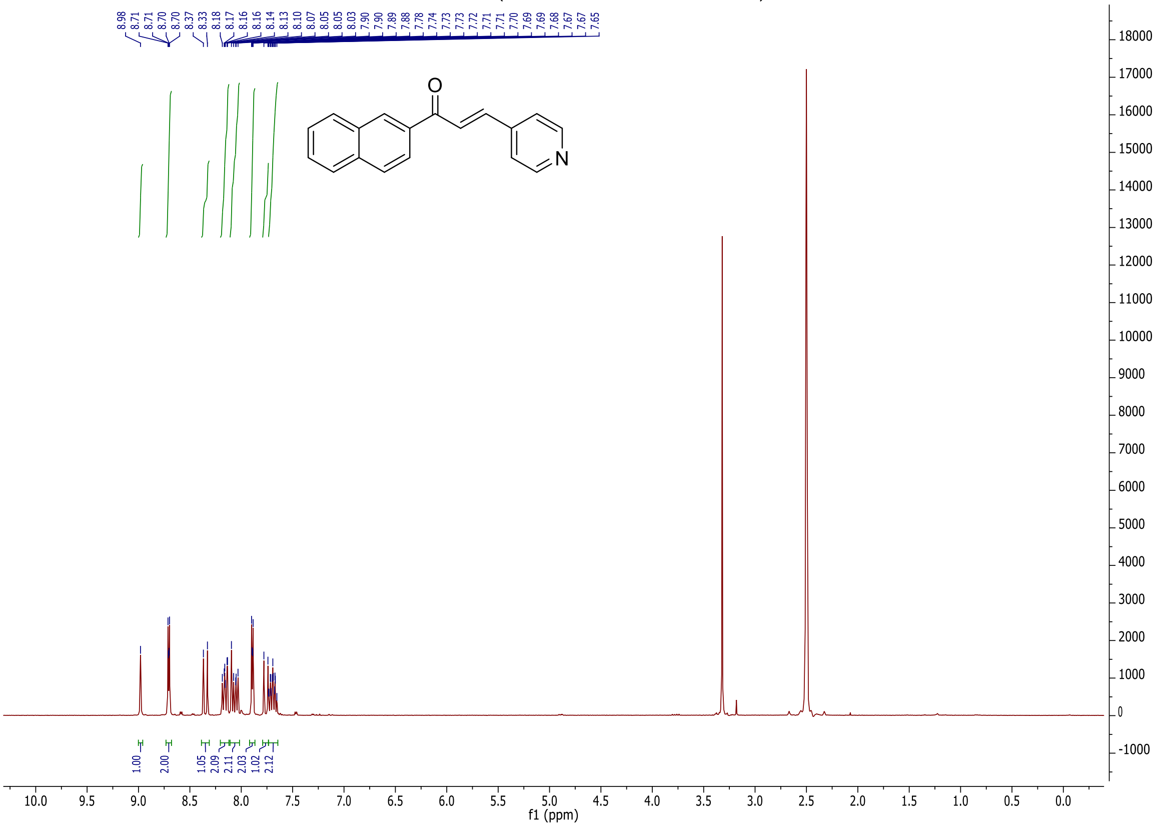

^{13}C-NMR (126 MHz, DMSO-d_6)

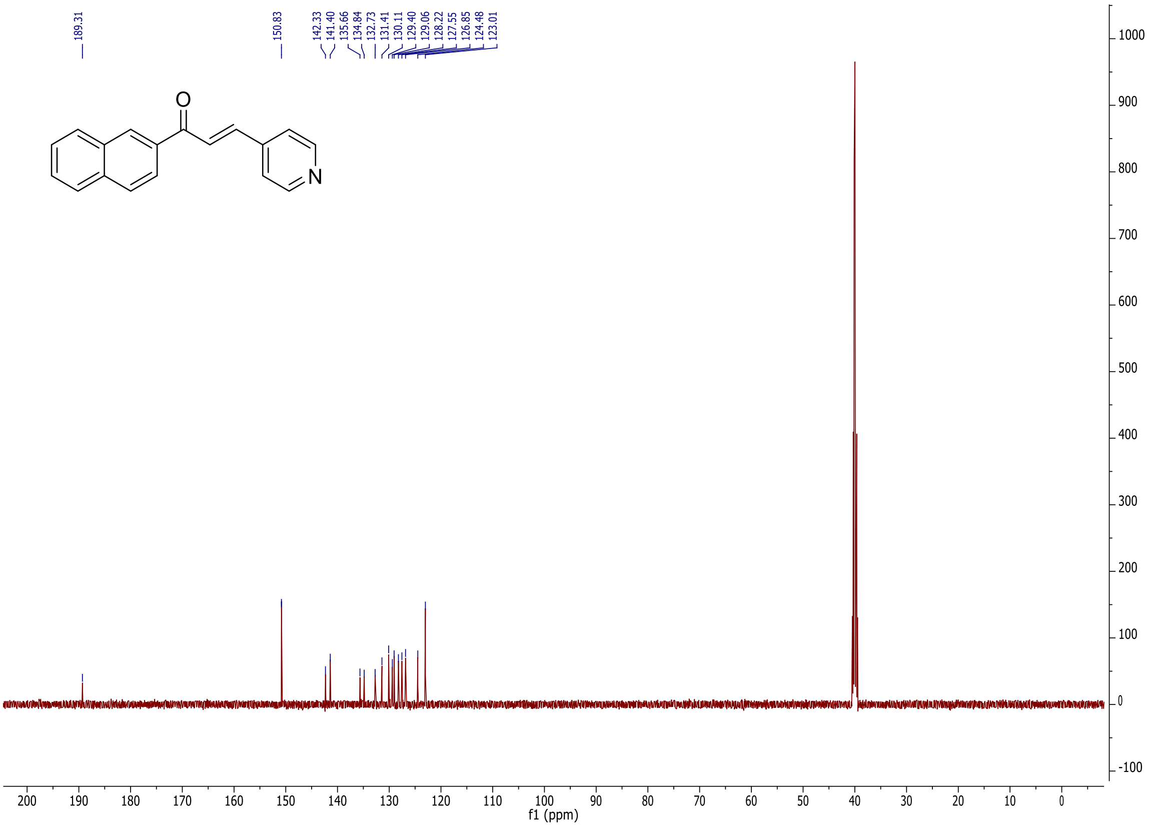

Compound 10

^{1}H-NMR (400 MHz, DMSO-d_6)

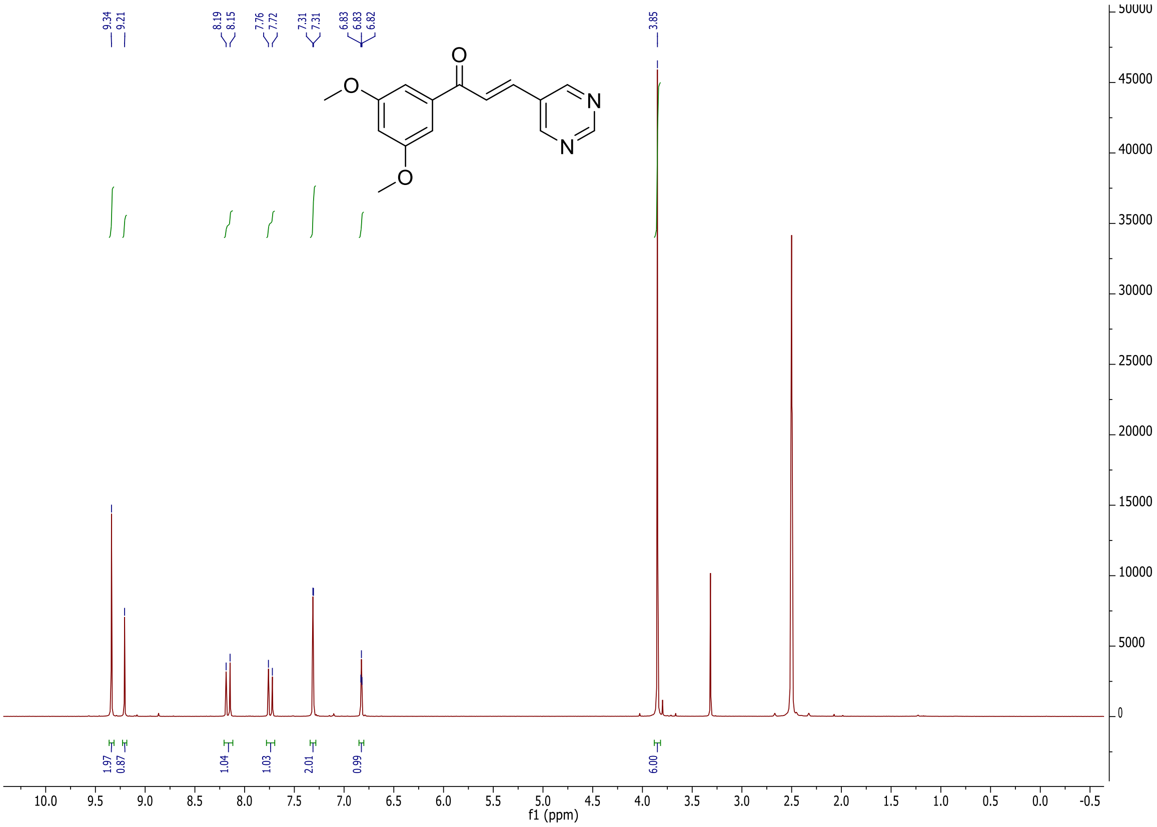

^{13}C-NMR (101 MHz, DMSO-d_6)

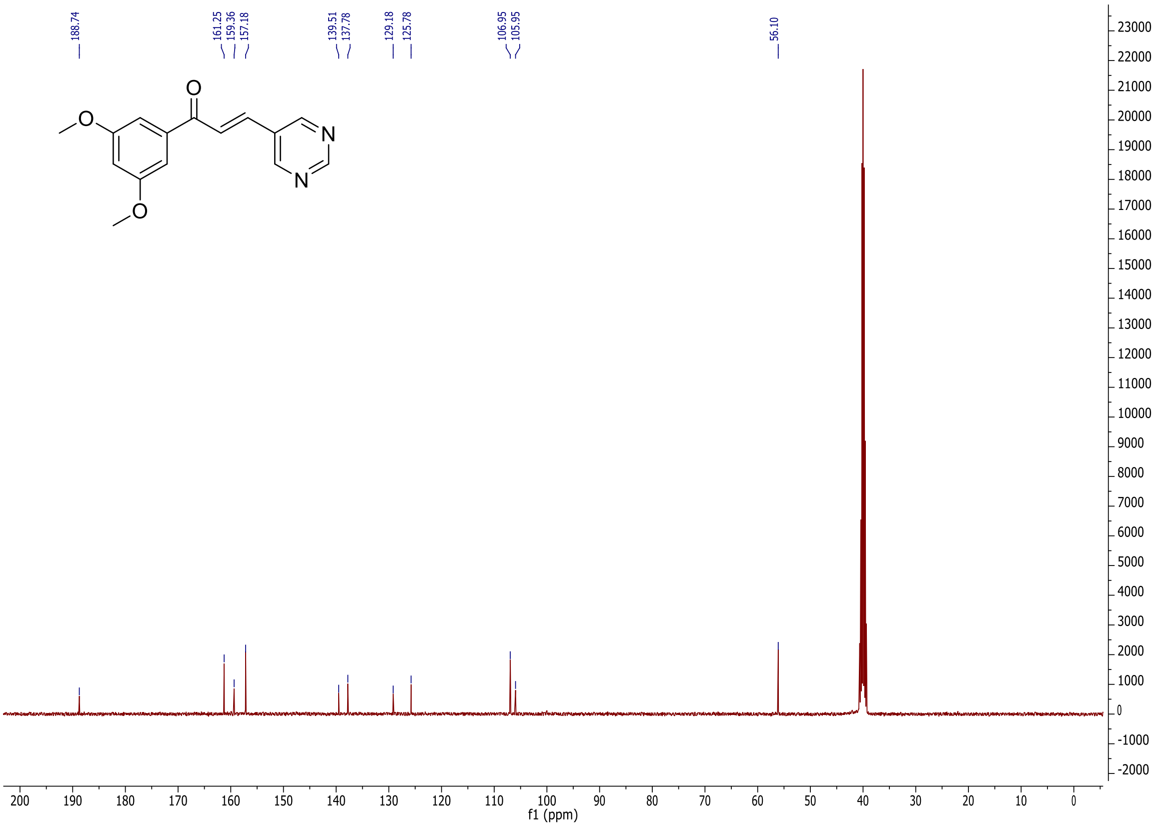

Compound 30

^{1}H-NMR (300 MHz, DMSO-d_6)

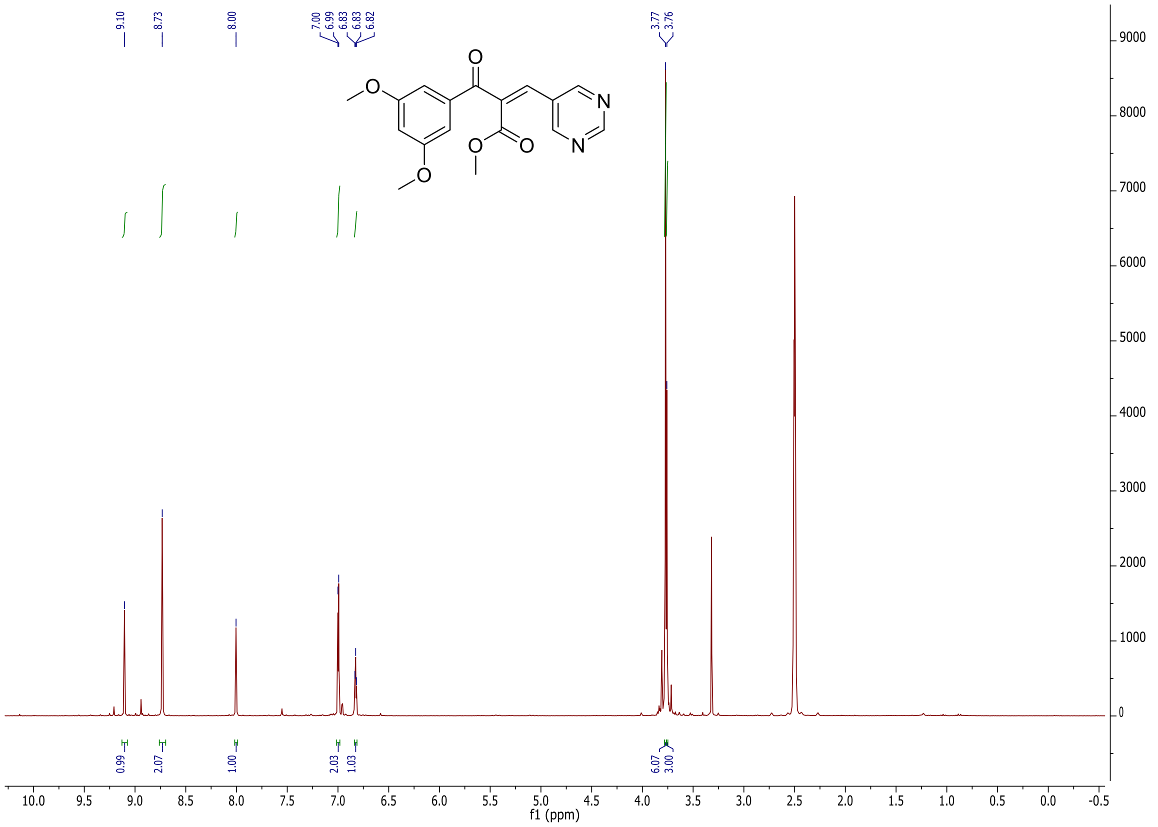

^{13}C-NMR (75 MHz, DMSO-d_6)

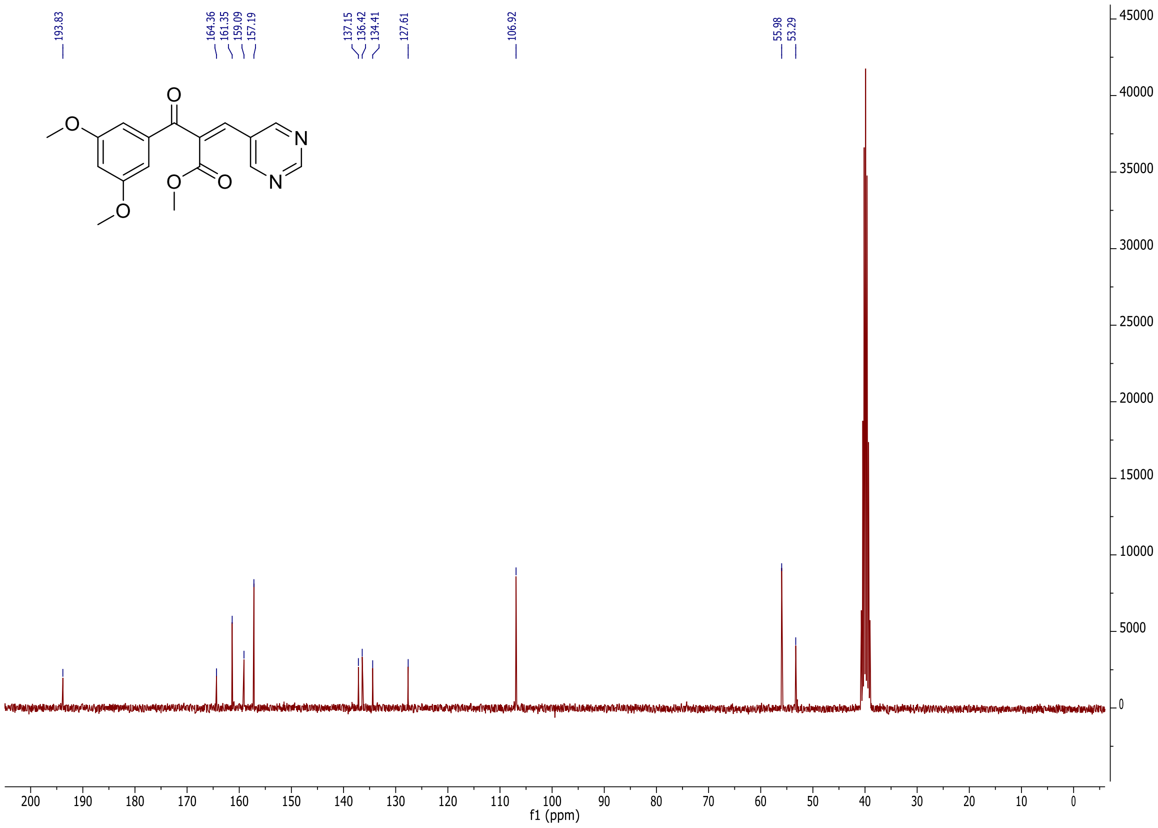

Compound 33

^{1}H-NMR (400 MHz, DMSO-d_6)

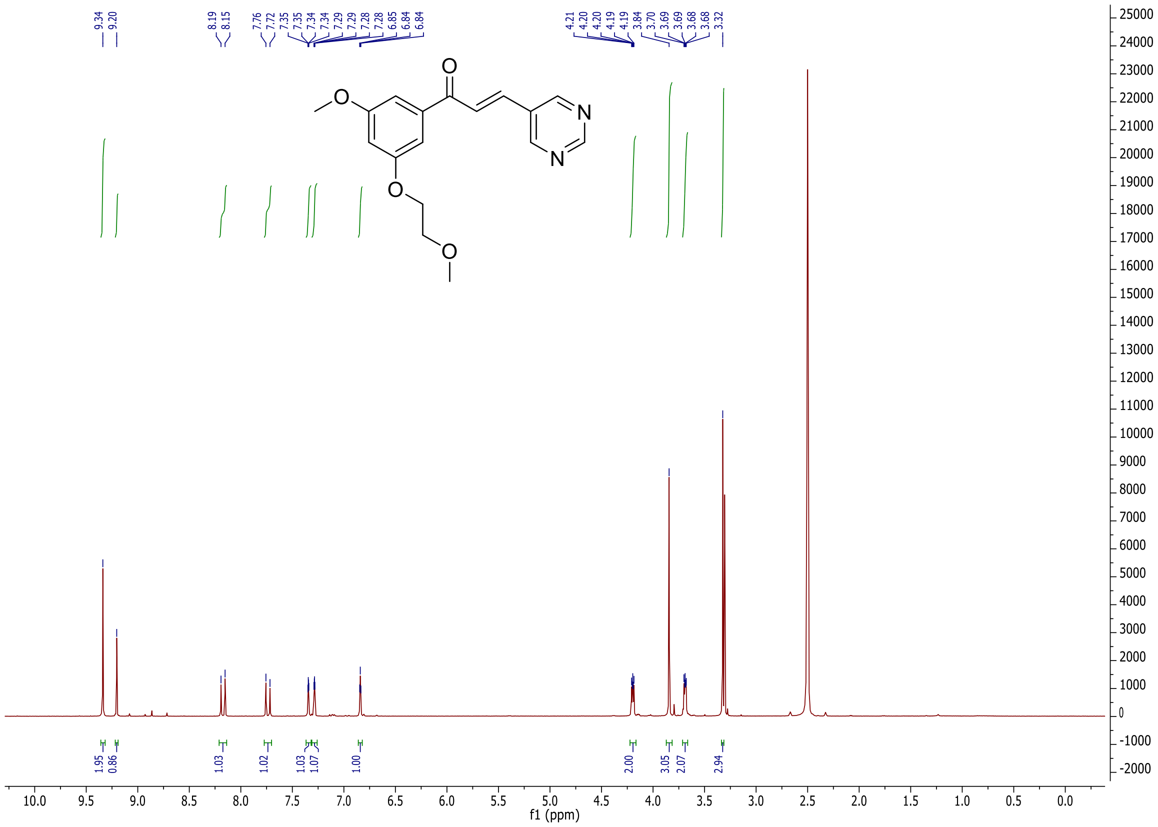

^{13}C-NMR (101 MHz, DMSO-d_6)

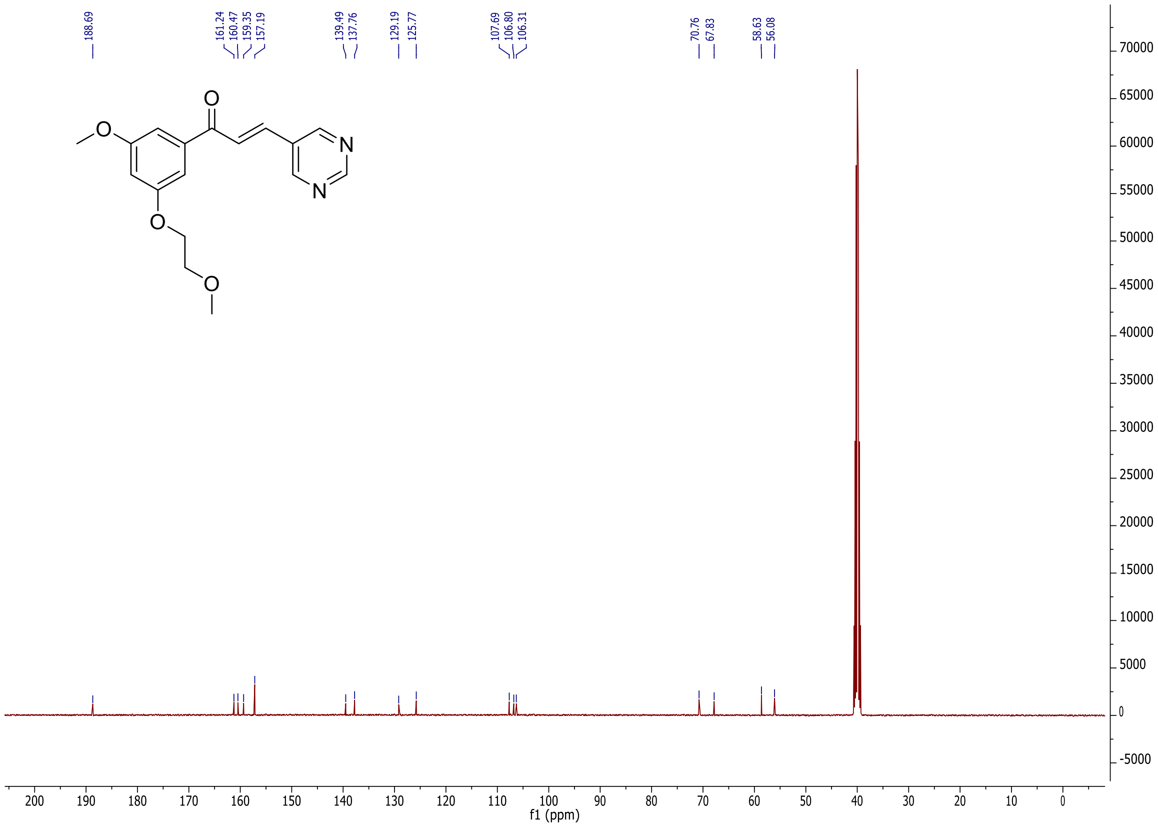

Compound 34

^{1}H-NMR (300 MHz, DMSO-d_6)

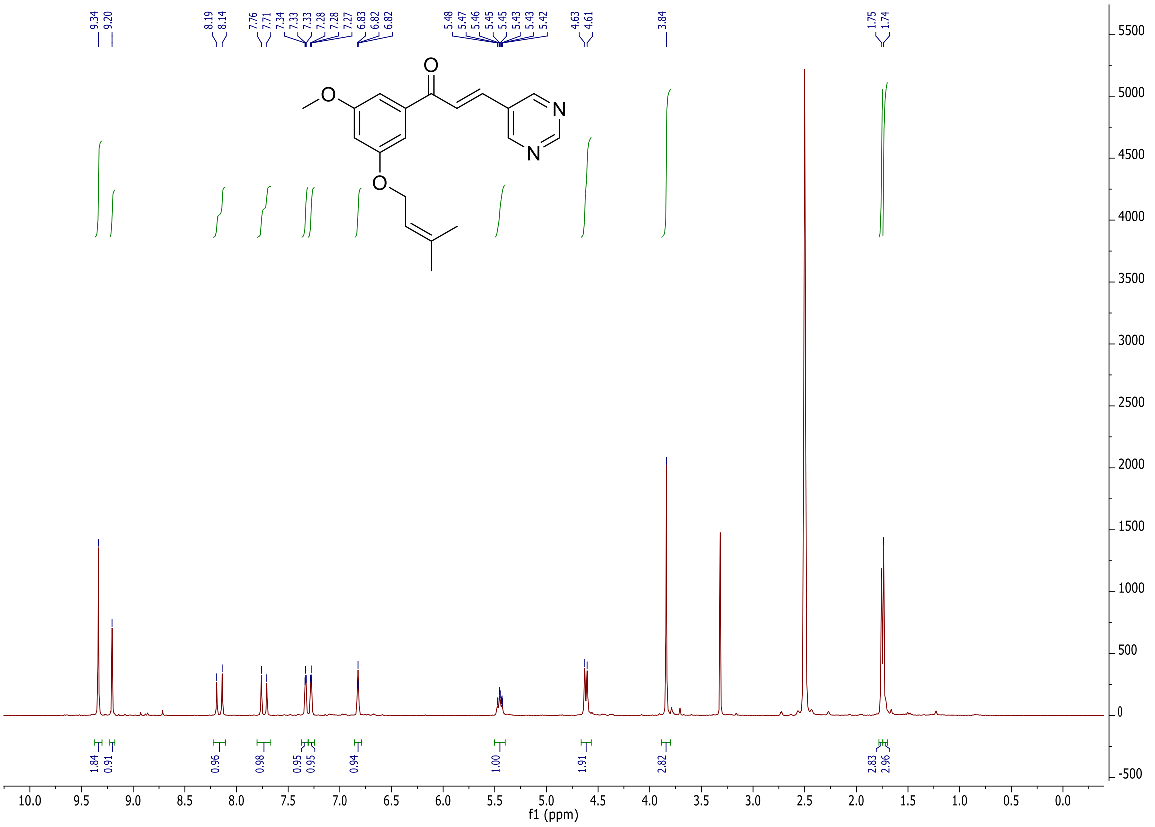

^{13}C-NMR (101 MHz, DMSO-d_6)

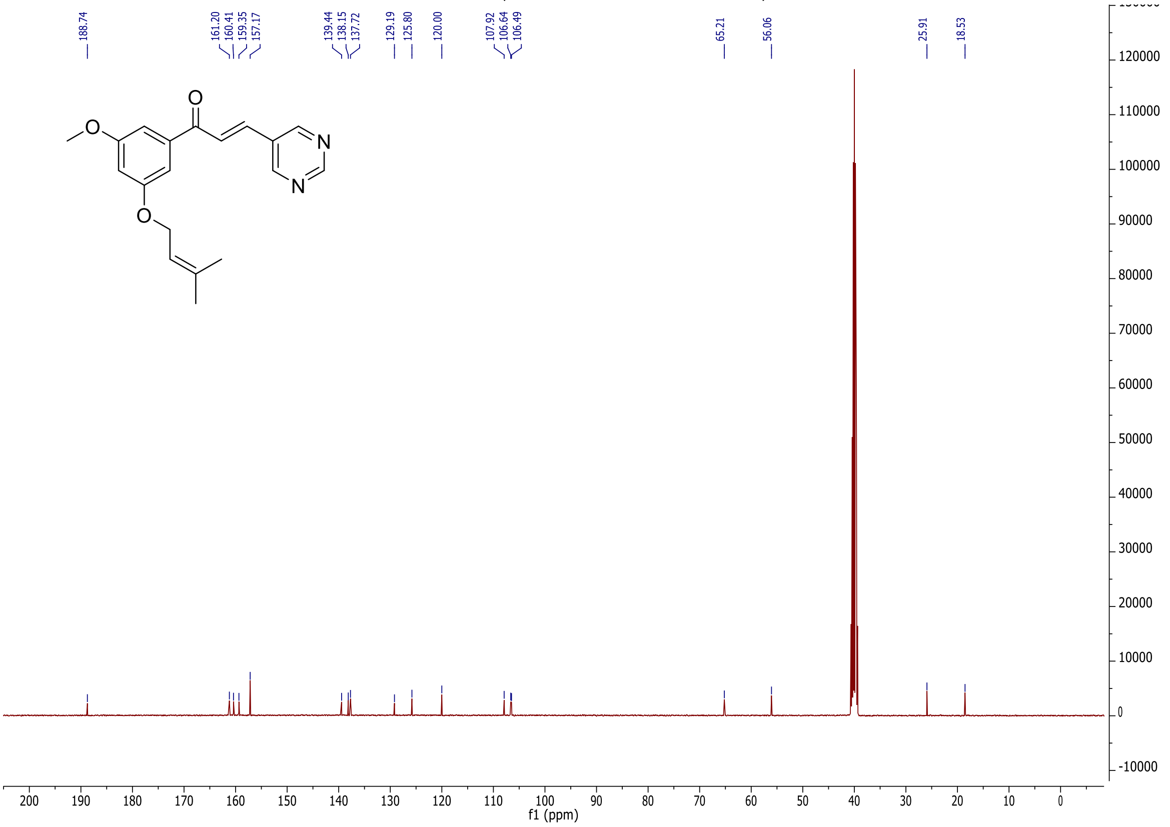

Supplement: Supplementary file 1 [file pharmaceuticals-14-01131-s001.zip › pharmaceuticals-1440036-supplementary.pdf]
